# Systematic review of physical and dosimetric criteria for managing CIEDs in radiotherapy: A medical physicist's perspective

**DOI:** 10.1002/acm2.70740

**Published:** 2026-08-16

**Authors:** Tiziana Malatesta, Agnese Barbareschi, Rita Alaimo, Emilio Mezzenga, Maria Grazia Brambilla, Anna Delana, Stefano Andreoli, Rita Consorti, Stefano Ren Kaiser, Marco Mapelli, Eugenia Moretti, Maria Mormile, Giulia Rambaldi Guidasci, Mara Severgnini, Lidia Strigari, Maria Daniela Falco

**Affiliations:** ^1^ Medical Physics Unit, Centro di Eccellenza Oncologia Radioterapica, Medica e Diagnostica per Immagini, Ospedale Isola Tiberina – Gemelli Isola Rome Italy; ^2^ Medical Physics Unit Ospedale S. Chiara Trento Italy; ^3^ Medical Physics Unit Ospedale di Perugia Perugia Italy; ^4^ Medical Physics Unit IRCCS Istituto Romagnolo per lo Studio dei Tumori (IRST) “Dino Amadori” Meldola Italy; ^5^ Medical Physics Department ASST Grande Ospedale Metropolitano Niguarda Milano Italy; ^6^ Medical Physics Unit ASST Papa Giovanni XXIII Bergamo Italy; ^7^ Medical Physics Unit Asl Roma 1 Roma Italy; ^8^ Medical Physics Department Azienda Sanitaria Universitaria Giuliano Isontina (ASUGI) Trieste Italy; ^9^ Medical Physics Unit Policlinico di Monza Monza Italy; ^10^ Medical Physics Unit ASUFC Udine Udine Italy; ^11^ UOC Radioterapia Ospedale del Mare ASL Napoli 1 Centro Napoli Italy; ^12^ Alma Mater Studiorum University of Bologna Bologna Italy; ^13^ Department of Medical Physics IRCCS Azienda Ospedaliero‐Universitaria di Bologna Bologna Italy; ^14^ UOSD di Fisica Sanitaria, ASL2 Lanciano‐Vasto‐Chieti Chieti Italy

## Abstract

**Aim:**

Current radiotherapy (RT) guidelines for Cardiac Implantable Electronic Devices (CIEDs) are largely based on clinical management strategies and retrospective reports. This systematic review adopts a medical physics perspectiv**e** to provide a critical, quantitative analysis of the technical evidence required for developing robust safety action plans.

**Materials and methods:**

Following the Preferred Reporting Items for Systematic Reviews and Meta‐Analyses (PRISMA) guidelines, a systematic search was conducted (January 2014–December 2024) across five major databases. Sixteen physicists from the Associazione Italiana di Fisica Medica e Sanitaria (AIFM) Working Group (WG) performed a double‐review and standardized data extraction, focusing on physics‐specific variables for the irradiation of Left Ventricular Assist Devices (LVADs), Pacemakers (PMs), and Implantable Cardioverter‐Defibrillator (ICD) devices. Data were grouped into five topics: photon/electron RT, hadron therapy, modeling RT effects (Treatment Planning System [TPS]/Monte Carlo [MC]), imaging, and specific considerations regarding Magnetic Resonance Imaging Linac (MRI‐Linac) systems. The Malfunction Rate (MR)—the percentage of devices with a clinically relevant malfunction—was calculated, and a Z‐test was carried out to highlight safer irradiation conditions. Additionally, a narrative synthesis was performed.

**Results:**

Of the 60 selected articles, 47 were co‐authored by physicists. The studies considered a total of 1769 real devices, while 13 studies reported on virtual devices (i.e., simulations, electronics, or measurements only). Reported irradiation beams included photons (encompassing Intra‐Operative RadioTherapy [IORT], Total Body Irradiation [TBI], and Flattening Filter Free [FFF] beams), electrons, protons, and carbon ions. Thirty‐seven manuscripts investigated CIED malfunctions, yielding an overall MR of 12.6%. The MR was significantly lower for devices irradiated under scatter conditions and when non‐neutron‐producing beams were used. Information on the entire RT process (imaging, dose calculation, planning, and irradiation) was analyzed, and a flowchart was developed to guide medical physicists in their daily clinical practice.

**Conclusion:**

The review concludes that the medical physicist's role is non‐negotiable in developing and implementing advanced planning and dosimetry strategies to prevent CIED malfunction and ensure patient safety.

## 1 INTRODUCTION

In 1958, the first implantable pacemaker (PM) was designed and introduced to treat cardiac arrhythmias,^1^ conditions causing palpitations, dizziness, syncope, and sudden cardiac arrest. With increasing life expectancy and an aging population, cardiovascular diseases and cancers have become the leading causes of death, necessitating the use of Cardiac Implantable Electronic Device (CIEDs), such as PMs for the treatment of bradyarrhythmia and Implantable Cardioverter‐Defibrillators (ICDs) for tachyarrhythmias.^2^, ^3^, ^4^ In patients with advanced heart failure awaiting transplantation, ICDs are often combined with Left Ventricular Assist Devices (LVADs), used as a bridge to transplantation or as destination therapy.^5^, ^6^

Over recent decades, CIEDs have evolved with reduced power consumption, enhanced reliability, and longer generator life.^7^ However, the Complementary Metal‐Oxide‐Semiconductor (CMOS) circuits in these devices are susceptible to ionizing and electromagnetic radiation, which can lead to device malfunction.^8^, ^9^, ^10^, ^11^, ^12^ In these cases, the failure mechanisms are primarily driven by three physical processes: (1) Total Ionizing Dose (TID), which causes permanent hardware damage through charge trapping in the silicon dioxide insulation layers; (2) Dose Rate (DR) effects, where transient photocurrents—particularly from high‐intensity modalities such as FFF beams—can mimic cardiac signals and lead to oversensing; and (3) Single Event Upset (SEU)—unpredictable software bit‐flips caused by stochastic nuclear interactions. Notably, while modern nanometric semiconductor nodes (e.g., 65 nm) have improved battery efficiency, they have also increased device sensitivity to SEUs.^13^, ^14^, ^15^, ^16^, ^17^, ^18^, ^19^, ^20^, ^21^, ^22^, ^23^, ^24^, ^25^, ^26^

This issue is particularly critical in the case of patients with CIEDs who require RT.^27^ Similarly, RT itself has undergone significant advancements, particularly with the introduction of modern TPS, image‐guided techniques, the increasing use of FFF beams, particle therapy, and Magnetic Resonance Imaging (MRI) guided radiotherapy. Consequently, the management of patients with CIEDs during RT has become increasingly relevant and complex.

In 1994, the American Association of Physicists in Medicine (AAPM) published Task Group (TG) Report No. 34,^13^ which provided guidelines for managing patients with PMs undergoing RT. Three recommendations remain relevant today: (1) avoid direct device irradiation; (2) estimate the device dose before RT; (3) if the cumulative dose to the PM is expected to exceed 2 Gy, the device should be interrogated before and monitored during treatment.^28^, ^29^, ^30^, ^31^, ^32^ Since then, technological progress in both CIEDs and RT techniques has emphasized the need for safe and accurate patient management.^32^, ^33^, ^34^, ^35^ These developments have highlighted the importance of careful RT planning and dose assessment,^28^ as well as the main risk factors for CIED malfunction, such as radiation energy, direct irradiation, RT technique, and cumulative dose.^29^

In response to these challenges, the AAPM TG 203^29^ proposed a risk classification system for CIED patients, categorizing patients into low, medium, and high‐risk groups based on cumulative CIED dose, pacing dependency, beam energy, and RT modality. This classification aims to guide clinicians in tailoring RT plans to minimize CIED‐related risks.^28^, ^36^, ^37^

In this scenario, a significant contribution was established by the German Society of Radiation Oncology together with the German Society of Cardiology through the Deutsche Gesellschaft für Radioonkologie/Deutsche Gesellschaft für Kardiologie (DEGRO/DGK) guidelines.^30^ Although published earlier than the AAPM TG‐203 report, the DEGRO/DGK recommendations introduced a highly effective management strategy that differs primarily in its clinical philosophy and planning restrictions. While both frameworks thoroughly address the risk of photoneutron generation and its role in causing stochastic SEUs, they propose different operational workflows. The AAPM TG‐203 manages high‐energy risks by assigning patients to higher risk categories, thereby intensifying the post‐planning interrogation and cardiac monitoring schedules. Conversely, the DEGRO/DGK guidelines focus on a stricter preventive approach, strongly restricting the use of neutron‐producing beams and enforcing the choice of low‐energy photons (≤6 MV) as a primary baseline requirement during treatment planning.

Even though several national and international guidelines exist,^28^, ^29^, ^30^, ^31^, ^38^, ^39^ they are principally clinical or management focused on CIEDs and the authors are mainly clinicians (i.e., radiotherapists, oncologists, cardiologists), showing a lack of the medical physics perspective. For this purpose and following the Preferred Reporting Items for Systematic Reviews and Meta‐Analysis (PRISMA) guidelines, this review aims to fill this gap by providing an updated synthesis of the last decade's literature across different databases, leading to a roadmap solution for the medical physics community to be considered during the planning and the course of radiotherapy treatment in patients with CIEDs (PMs/ICDs/LVADs).

## 2 STUDY SELECTION

### 2.1 Systematic review search strategy

Sixteen members from the AIFM WG on CIEDs conducted this systematic review according to PRISMA guidelines using structured search terms (see http://prisma‐statement.org/). Five members acted as project coordinators (PCs), and the remaining 11 were reviewers. The comprehensive literature search, guided by the Population, Intervention, Comparison, and Outcome (PICO) framework, spanned five major databases (PubMed/MEDLINE, Google Scholar, Embase, Scopus, and Web of Science) and was restricted to articles published between January 2014 and December 2024.

### 2.2 Study selection

Based on the PICO approach (P = PMs, ICDs, LVADs; I = Radiotherapy; C = Management of CIEDs from a physical point of view; O = Guidance/Recommendations for physicists), inclusion criteria comprised: (1) studies on PMs, ICDs, or LVADs; (2) English, peer‐reviewed manuscripts; and (3) case reports (of relevance) or clinical studies of specific RT techniques or focuses (e.g., LVAD, hadrons, FFF beams, Stereotactic Body Radiation Therapy (SBRT) techniques, MRI‐guided radiotherapy), as well as in vitro studies.

Exclusion criteria considered case reports and clinical studies not included in the previous list, guidelines, reviews, meta‐analyses, non‐RT studies, and publications lacking a technical focus.

Reviewers could, however, take into account a small number of technically relevant publications even if the “full article” was unavailable.

### 2.3 Data extraction

Publications retrieved from the database search were screened by the PCs based on title and abstract. A standardized data extraction sheet (see Supplementary Table S1) was developed, validated, and refined through pilot testing. Extracted information included general details (title, year, journal) and study characteristics (in vitro/in vivo design, radiation delivery, imaging modality, objectives). Additional technical data device number and type (PM, ICD, or LVAD), irradiation methodology (e.g., skin distance, direct/scattered exposure), TPS parameters, and malfunction were systematically collected. Each study was independently reviewed by two WG members; MR discrepancies were resolved by a third reviewer.

### 2.4 Data synthesis and analysis

Data were classified into two main device groups: LVADs and PM/ICDs. The latter, being more extensively studied, were further divided into five topics: (1) photon and electron radiation therapy (in vitro, in vivo studies), (2) hadron therapy (in vitro, in vivo studies), (3) modelling RT effects (TPS accuracy (comparison between calculated and measured doses), MC simulation), (4) imaging in RT, and (5) MRI‐guided radiotherapy.

A graphical summary is provided to classify the reviewed articles by topic, device type (PM, ICD, LVAD), irradiation condition (in‐field/out‐of‐field, in vivo/in vitro), beam type, and dose. Studies were also categorized according to their primary focus—device functionality and malfunctions versus other technical aims.

For papers addressing device performance, key physical variables influencing malfunctions were analyzed: radiation type (photons, electrons, protons, ions), irradiation condition (in‐field/out‐of‐field), beam energy (for photons), DR for FFF beams, and total device dose. Reported malfunctions were categorized as software or hardware; permanent (those that do not resolve spontaneously) or transient; online or post‐irradiation; and CR or not, based on international consensus guidelines and established literature.^10^, ^11^, ^12^, ^40^ In particular, CR malfunctions were defined as events directly impacting patient safety or therapy delivery, including: pacing inhibition, inappropriate ICD therapies (shocks or anti‐tachycardia pacing), permanent hardware failure, and Power‐on Resets resulting in the loss of patient‐specific programming and deactivation of ICD therapies. Not CR malfunctions were defined as minor technical anomalies that do not compromise immediate therapeutic efficacy, such as transient telemetry communication loss during beam delivery, loss of non‐therapeutic diagnostic data, or minor variations in electrical lead parameters (pacing thresholds and impedances) remaining within standard safety margins and not requiring clinical reprogramming. The MR, defined as the percentage of devices showing CR malfunctions among CIEDs in articles investigating device malfunction, was calculated. MR was also calculated only considering transient malfunctions to evaluate the influence of FFF beams.

Differences in MR across physical variables were assessed using the Z‐test (*α* = 0.05) or, for small samples, Fisher's exact test (*α* = 0.05).

The findings were incorporated into a structured, clinically‐oriented flowchart to guide medical physicists in making suitable choices and considering key aspects when optimizing treatments for patients with CIEDs.

Emerging patterns and common findings were identified and discussed.

The review protocol was registered in PROSPERO, the International Prospective Register of Systematic Reviews (CRD42024550524).

### 2.5 Literature synthesis and findings

Following the procedure, relevant literature was compiled and distilled. Given the heterogeneity of the issues investigated, a narrative synthesis was performed to summarize findings for each topic. To improve clarity, a single summary table was created that consolidates all bibliographical information, highlighting the physical and dosimetric aspects of each topic covered.

## 3 MAIN FINDINGS FROM SELECTED LITERATURE

### 3.1 Statistic overview

**FIGURE 1:**
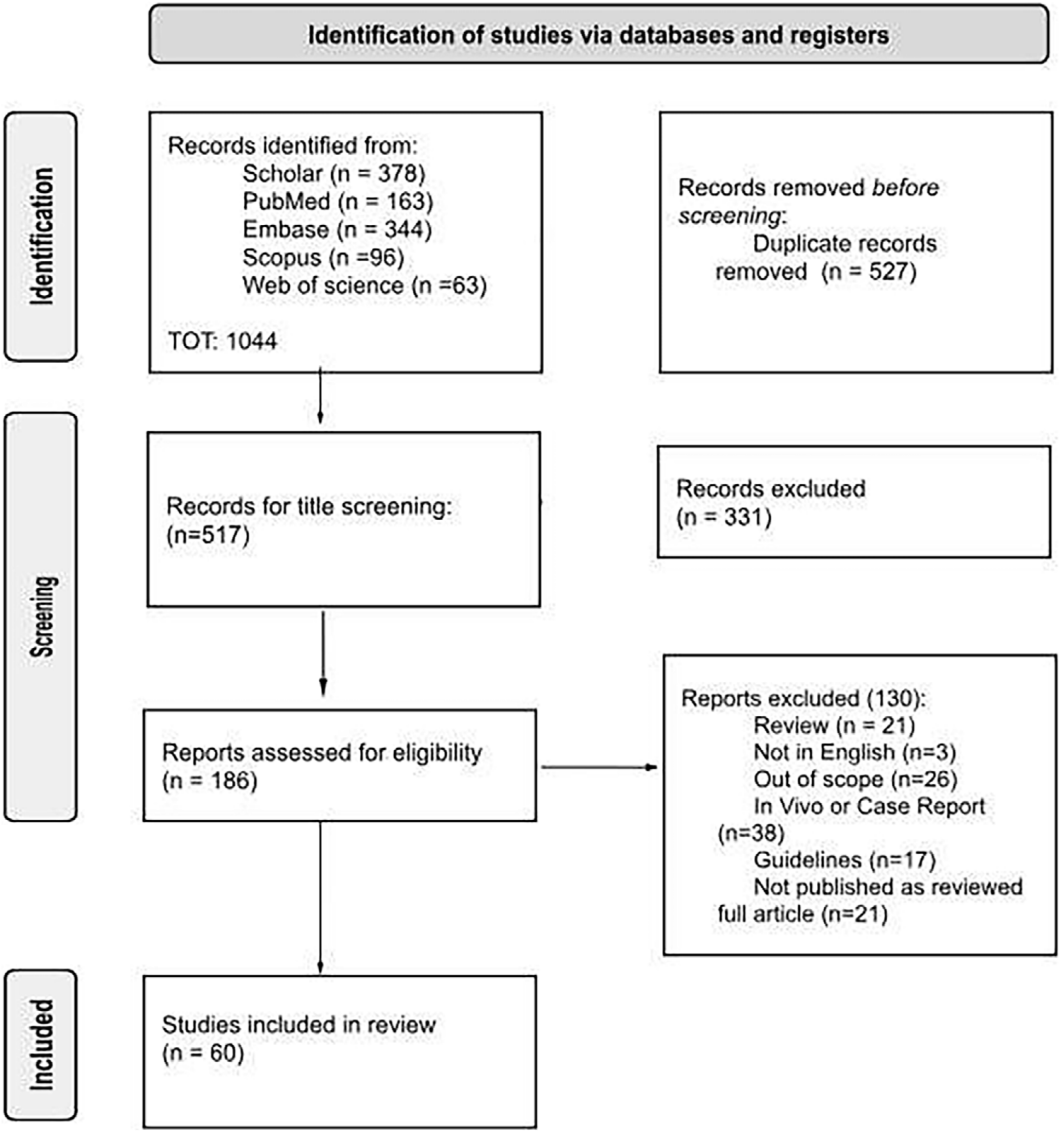
PRISMA flow diagram for selecting relevant papers.

**FIGURE 2:**
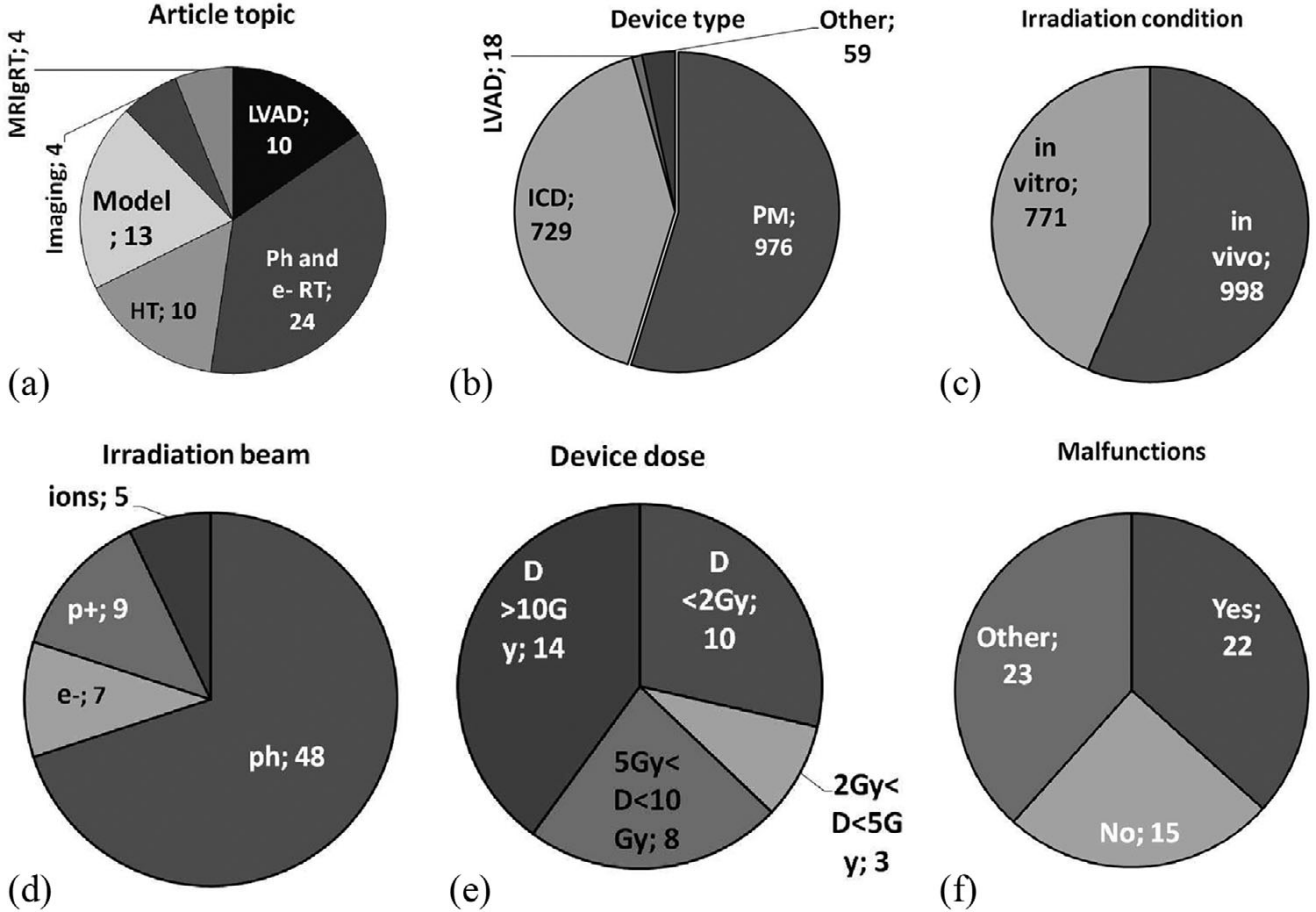
(a) Number of articles per topic considered, categorized by topic: LVAD, PM/ICD Devices and Photon/Electron Therapy: (Ph and e‐ RT), PM/ICD Devices and Hadron therapy (HT), Modelling RT effects (Model), Imaging, and MRI guided radiotherapy (MRIgRT). (b) Types of CIEDs considered in the articles (number of CIEDs). “Others” refers to cases where the device type was not explicitly stated, or where CIED surrogates or simulations were used. (c) Number of CIEDs irradiated in vivo and in vitro. (d) Irradiation beam: photons (ph), electrons (e−), protons (p+), ions used; (number of articles). (e) Maximum reported device dose in articles, when explicitly stated (number of articles). (f) Number of articles investigating device malfunction that reported device malfunction (Yes), those that did not report any malfunction (No), and articles with a different focus (Other).

Following initial screening, 60 articles were included (Figure 1), with medical physicists among the authors in 47 of them. The number of articles for each topic was: LVAD: 10, and (for PM/ICD) photon and electron RT: 24, hadron therapy: 10, modeling RT effects: 13, imaging: 4, and MRI guided radiotherapy: 4 (Figure 2A). Five studies handled two topics.^41^, ^42^, ^43^, ^44^, ^45^

A total of 1782 CIEDs were found (Figure 2B), including 1769 real devices (976 PMs, 729 ICDs, 18 LVADs, and 46 unspecified whether PM or ICD). Additionally, 13 articles investigated virtual devices: five performed MC simulation and eight evaluated the dose at the typical device location or irradiated Field‐Programmable Gate Array (FPGA) circuits (FPGA are integrated circuits widely used in electronic devices, including CIEDs, and contain numerous radiation‐sensitive memory units). Device manufacturers included: Medtronic (Dublin, UK), Biotronik (Berlin, Germany), St. Jude Medical (St. Paul, USA) now Abbott (Chicago, USA), Sorin Group (Milan, Italy), Guidant (Indianapolis, USA), Medico (Padova, Italy), and Vitatron (Maastricht, The Netherlands) for PMs/ICDs; Abbott (Chicago, USA), Medtronic (Dublin, UK), Jarvik (New York, USA), and Novacor (Oakland, USA) for LVADs.

Of the real devices, 56.4% (998) were irradiated in vivo, while 771 were irradiated in vitro (Figure 2C). CIEDs were irradiated both within the primary beams (26 articles) and in scatter radiation (44 articles).

The majority of articles (48) studied photon beams, nine of which used FFF beams (DRs between 400 MU/min and 2500 MU/min) and four considered MRI‐Linacs. Seven studies considered electrons, 12 studies considered hadrons (seven proton beams, three heavy ions, and two utilizing both) of which five used active scanning techniques, five passive scattering, and two did not declare it. Ten articles considered more than one beam type (mostly photons and electrons or protons and ions), and one article did not declare the beam (Figure 2D). Thirty‐two articles used neutron‐producing beams (i.e., photon energies > 6 MV, proton, or ion beams). Energies and techniques evaluated are detailed in Table 1.

**TABLE 1:** Number of articles categorized by beam type and energy.

Irradiation beams	Energies	No. of articles	Techniques
Photons WFF*	< = 6MV	14	Direct beam, 3DCRT, IMRT, VMAT, SBRT
	> 6MV	17	
	N.A.	9	
**Photons FFF**	< = 6MV	2	
	>6MV	4	
**Electrons**	6–18 MeV^**^	7	IORT, direct beam
**Protons**	115–235 MeV^**^	5	Active spot scanning
		3	Passive scattering
		1	NA
**Ions**	140–400 MeV^**^	1	Active raster scanning
		3	passive scattering
		1	NA

*Note*: Many articles consider multiple irradiation beams.

* For this analysis, unless FFF (Flattening Filter Free) beams are explicitly mentioned, all photon beams are assumed to be WFF (With Flattening Filter).

** When explicitly stated.

NA = Not available.

The device dose was reported by 35 articles (Figure 2E) and the maximum dose varied significantly across the studies. In vivo dosimetry was performed in 21 studies, and seven included real‐time monitoring. Only 11 articles specified the setup imaging type (kilovolt Cone Beam Computed Tomography (kV‐CBCT), kilovolt (kV), or Megavolt (MV) images, MRI), with one estimating the dose.^46^

#### 3.1.1 Device malfunctions statistics

**FIGURE 3:**
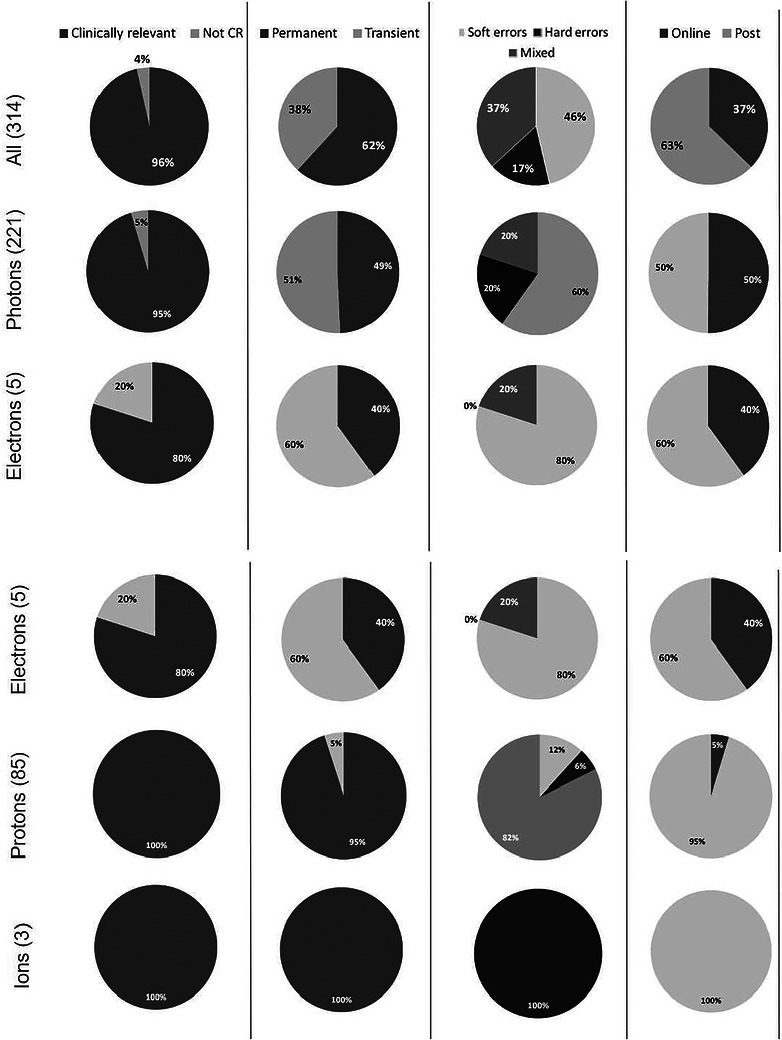
Malfunctioning events were categorized based on the following attributes: clinical significance (Clinically Relevant CR or not); nature of the effect (permanent or transient); origin of the error (software, hardware, or mixed); and timing of occurrence (online or post‐irradiation). The events were first analyzed in aggregate and subsequently evaluated for each beam type individually. In brackets the number of errors found for each beam type.

Device malfunctions were investigated in 37 papers, covering 1680 PMs or ICDs, 18 LVADs and one virtual CIED. Of these, 22 reported anomalies (Figure 2F) affecting 214 devices (i.e., 12.6%) and accounting for 315 malfunction events. Events were classified as CR/non‐CR, permanent/transient, soft/hard (or mixed), and online/post irradiation. Figure 3 shows the distribution of these events per beam type.

The MR was significantly lower for PMs (9.3%) than for ICDs (17.1%) while for LVADs it was 5.6%.

The only LVAD malfunction occurred after direct proton irradiation of the pump. No statistical analysis was possible due to insufficient data.

The following analysis focuses only on PMs and ICDs. The MR was significantly lower for devices irradiated with photon beams (12.2%) compared to those irradiated with hadrons (18.0%). Only one study^47^ reported CR malfunctions with an electron beam: it was a CIED under the primary beam receiving more than 5 Gy.

As shown in Figure 3, all hadron‐induced malfunctions were CR, mostly permanent and post irradiation.

For photon beams, MR was significantly lower when the cumulative dose was < 5 Gy (9.2%) than when it was ≥5 Gy (16.4%). Device position was also critical: MR in scattered radiation was 6.0%, and 24.2% under the primary beam.

Beam energy influenced MR: 6 MV beams showed lower MR than higher energies, both in scatter (2.0% vs. 14.8%) and in‐field (19.8% vs. 68.2%) conditions. For 6 MV beams, flattened (With Flattening Filter; WFF) beams yielded lower MR than FFF (scatter: 0.7% vs. 9.6%; primary: 18.0% vs. 46.4%), with no significant difference at higher energies.

If considering only transient malfunctions, the MR was significantly higher for FFF than WFF (scatter: 9.09% vs. 1.1%; primary: 36.8% vs. 12.4%).

Devices irradiated with hadron beams were all in scatter conditions at low doses (CIED‐target distance > 4 cm), therefore no statistical analysis was possible.

### 3.2 Literature review

In this section, the articles selected during the reference period are discussed, divided by type of device (LVAD or PM/ICD) and by topic; their main information is reported in Table 2.

**TABLE 2:** Summary of main reviewed literature. The papers reviewed are categorized by device and topic, as follows: LVAD Devices (A); PM/ICD Devices and Photon/Electron Therapy: In Vitro Studies (B) and In Vivo Studies (C); PM/ICD Devices and Hadron Therapy (D); PM/ICD Devices and Modeling RT Effects: TPS Accuracy (calculated vs. measured/simulated doses) (E) and Monte Carlo (MC) Simulation (F); PM/ICD Devices and Imaging in RT (G); MRI‐related considerations and MR‐guided Radiotherapy (H).

(A) LVAD devices										
Ref	Author/Year	Study Design (in vivo/in vitro)	# LVADs Real (R) – Virtual (V)^*^	Device Manufacturer	Radiation Type/Energy	Technique	Irradiation details (set up “in vitro”/target “in vivo studies/dose prescription)	CIED Position in‐field/out‐of‐field	Dose to CIED	Malfunction Type/Other outcome
44	Sindhu et al. /2022	In vitro	1 R	Abbott (HeartMate 3)	Protons/SOBP range = 100 mm (secondary neutrons present)	Open field (pencil beam scanning)	CIED in the middle of SOBP (width = 6 cm), @ 5 cm depth/70 and 75 GyE different fractionations and continuously delivered	In‐field	Pump: 70 GyE; driveline: 70 and 75 GyE	Communication lost between pump and controller; flow alarm at 30 GyE (continuous irradiation)
48	Oishi et al. /2022	In vivo, in vitro	2 R	Abbott (HeartMate II)	Photons/6 MV	In vivo and 1st experiment: NA 2nd experiment: open field	In vivo: H&N; In vitro: 1st experiment: same as the patient, in an anthropomorphic phantom; 2nd experiment: CIED under a cushion with tissue‐equivalent beads; measures with dosimeter chips/50 Gy in 1 fr	In vivo and in vitro 1st experiment: out‐of‐field; In vitro 2nd experiment: in‐field	In vitro 1st: pump: 4,49–7,86 cGy; driveline: 0,57–6,34 cGy; system controller: 0,41–0,66 cGy; power cable: 0.13–0.38 cGy; power module: 0,08–0,14 cGy; in vitro 2nd: system controller: 50 Gy	No malfunctions observed, even with direct irradiation at high doses
49	Ostertag‐Hill et al. /2018	In vivo	2 R	LVAD Medtronic (HeartWare); ICD Medtronic (Protecta XT CRT‐D)	Photons/6 MV	SBRT IMRT	Left lung lower lobe/50 Gy in 5 fr every other day; measurements with OSL dosimeters	Out‐of‐field	Calculated dose to LVAD: mean 45 cGy, max 698 cGy;	No malfunctions observed
50	Emerson et al. /2016	In vivo	4 R	LVADs Abbott (HeartMate II); ICD (NA)	Photons/6 and 15 MV (secondary neutrons present for 15 MV)	Patient 1: SBRT Patient 2 and 3: technique not reported	Patient 1: lung lower lobe/54 Gy in 3 fr with 6MV; Patient 2 palliative: C5‐T3/30 Gy, T6‐T11/20 Gy, pelvis/30 Gy, T9‐L4:30 Gy with 6 and 15 MV; Patient 3: gastroesophageal tumor and lymph nodes/50,4 Gy with 15 MV	Out‐of‐field	Patient 1: mean 9.6 cGy, max 61 cGy; patient 2: mean 1423 cGy, max 2450 cGy; patient 3: mean 1922 cGy max 4900 cGy	No malfunctions observed. Shielding the external system controller and external power source with a polyethylene boron shield is suggested.
51	Webster et al. /2024	In vivo/in vitro	1 R (in vivo study); 1 V (in vitro study)	Abbott (HeartMate 3)	Photons/16 MV (secondary neutrons present)	Open field (with internal shields)	Whole body/4 Gy in 2 fractions; measurements with OLS dosimeters for photons; additional measurements in solid water phantom with ionization chamber to acquire relative dose information; neutron dose estimated with survey meter.	Pump: in‐field (shielded with Cerrobend block); Controller: out‐of‐field in 1 cm acrylic box	Pump: 120 cGy, controller: 91,5–135,3 cGy neutron does:0,46 cGy for the entire treatment	TBI did not cause malfunctioning on the LVAD. OSLD measurements showed good agreement with the expected dose. The neutron dose was not affected by the lead compensators,
53	Scobioala et al. /2015	In vivo	3 R	LVAD Novacor (model NA);PM St. Jude Medical (Accent); ICD Matesa (Atlas II VR)	Photons/6 and 15 MV (secondary neutrons present)	3DCRT (6 and 15 MV) and SBRT helical Tomotherapy (6 MV)	Left main bronchus/25.2 Gy in 14 fractions (3DCRT) + 35 Gy in 5 fractions (SBRT); measurement with TLD; phantom measurement before treatment	LVAD and PM out‐of‐field; ICD partially in‐field	LVAD: mean 2,31, max 5,38 Gy; PM: 1,13 Gy, max 2,74 Gy; ICD: 5,55 Gy, max 15,58 Gy	No malfunctions observed
54	Gimenez De Lorenzo et al. /2020	In vitro	1 R	Jarvik Heart (Jarvik 2000)	Photons/10 MV (secondary neutrons present)	Open field	Field size:10 × 10 cm^2^, SSD:100 cm; depth:10 cm; in a water box with additional water‐equivalent stacks/6,8 Gy in 1 fraction	In‐field	Mean 660 cGy, max 820 cGy, min 420 cGy	TPS underestimation (Elekta Oncentra Masterplan) of the absorbed dose from the pump due to a bad modelling of high‐Z pump components
94	Benali et al. /2023	In vivo	5 R	Abbott (HeartMate III, HeartMate II) Medtronic (HeartWare)	Photons/NA	SBRT	Right or left ventricle/25 Gy in 1 fraction	NA (LVAD components can be deemed close to the target)	NA (2 Gy constraint used in 1 case)	Cardiac SBRT can be considered a promising and non‐invasive treatment in refractory ventricular arrhythmias management
95	Spano et al. /2020	In vivo	2 R	LVADAbbott (HeartMate III); CRT‐D (NA)	Photons/6 MV	1 Coplanar arc and 5 coplanar IMRT fields	Left lung middle lobe/50 Gy in 5 fractions; (measurement after 1st fraction with a pinpoint ionization chamber inside a brass build‐up cap);	LVAD pump and CRT‐D out‐of‐field; LVAD controller and driveline as far away as possible and shielded with 0.35 mm Pb	LVAD: pump: mean 8 cGy, max 29 cGy; controller: max 0,441 cGy driveline: mean 11 cGy, max 34 cGy; CRT‐D:mean 6 cGy, max 69 cGy; wires: mean 184 cGy, max 3319 cGy	No malfunctions observed
96	Schumer et al. /2022	In vivo	2 R	LVAD Abbott (HeartMate II); ICD (NA)	Protons/NA (secondary neutrons present)	NA	Left lung upper lobe and satellite lesion in the left lung lower lobe/60 Gy in 30 fractions;	Out‐of‐field; controller and driveline neither shielded nor dislocated	NA	No malfunctions observed

(B) In vitro studies										
Ref	Author/ Year	#CIEDs real (R)‐ virtual (V)*	Device Manufacturer	Radiation Type/Energy	Technique	TPS/algorithm	Irradiation details (set up “in vitro” /target “in vivo” studies/dose prescription)	CIED Position in‐field/Out‐of‐field	Dose to CIED (TPS/in vivo dosimetry/simulation) *	Malfunctions Type
14	Nakamura et al./ 2021	1 R / PM	Medtronic	Photons/10 MV FFF	Open field	NA	SAD = 100 cm; field 10 × 10cm^2^; device depth = 2,2 cm	In‐field	Up to 140Gy	Mild battery depletion (10Gy) Pacing‐output voltage change > 25% (122Gy) Loss of telemetry (134Gy) Lead impedance unreadable (134 Gy)
16	Mollerus et al./ 2014	4 CRT; 4 ICD (R)	Boston Scientific	Photons/6 MV	Open field	NA	SSD = 100cm; field size: 10cm × 10cm; device located at build‐up	In‐field	Escalating doses were administered over 8 days: 0.01, 0.1, 1, 10, 30, 30, 30, and 30 Gy	Failure to deliver shock therapy Significant fall in pacing impedance Increase in low‐energy shock impedance Battery status change to end of life Memory Class faults due to system‐on‐chip single bit memory corruption Telemetry system fault due to authentication failure
17	Zaremba et al./ 2014	10 PM: 2 ICD (R)	Biotronik; Boston Scientific; Medtronic; Sorin; St Jude Medical	Photons/6 MV, 18 MV	Open field	Eclipse v 10.0	SSD = 100cm; field size: 10 × 10cm^2^ (for PM) & 15 × 15cm^2^ (for ICD); device located at build‐up;	In‐field	2 Gy/day for 5 days, up to a cumulative dose of 150 Gy per device.	Resets after 30, 42, 80, 100, 120, and 150 Gy ‐18MV No telemetry after 150 Gy – 6MV; RRT/ERI detected after 150 Gy – 18MV Error after 150 Gy – 18MV Loss of patient data after 44 Gy – 18MV
18	Zecchin et al./ 2016	34 PM; 25 ICD (R)	Biotronik Boston Scientific St Jude Medical Sorin Group	Photons/15 MV	Open field	NA	NA	Out‐of‐field	70 Gy/1 fr	Electrical Reset Interrogation Impossible Backup in VVI mode Magnet deactivation Change of programmation
28	Zecchin et al./ 2018	19 R /ICD	Boston Scientific, Medtronic, Sorin, Biotronik, St. Jude Medical	Photons/6 MV	Open field	NA	NA	In‐field	Increasing cumulative photon doses: 0.5 Gy, 1 Gy, 2 Gy (at 0.17 Gy/min); 3 Gy, 5 Gy, and 10 Gy (at 0.85 Gy/min)	None
40	Falco et al./ 2022	210 R/ 145 PM; 65 ICD	St. Jude Medical, Medtronic, Boston Scientific, Biotronik, Medico, Sorin/Ela	Photons/6 MV	Open field	Eclipse Oncentra Masterplan Pinnacle	SSD = 96cm, Opposed 20 × 20 cm^2^ beams, CIED positioned at the center of a 20 × 23 cm^2^ basin filled with 2 L of physiologic solution (depths 3cm) placed on a 30 × 30 × 5cm^3^ RW3 solid phantom	In‐field	2, 5 and 10 Gy	Noise‐related interference Pacing inhibition Inappropriate detections (AF/VT/VF) Soft reset/ non‐reprogrammable backup Abnormal battery depletion/unexpected switch to E.R.I. Complete loss of telemetry
47	Baehr et al./ 2021	3 PM; 18 ICD (R)	Medtronic, St. Jude Medical, Biotronik, Guidant, Sorin	Photons/6 MV, electrons/6 MeV and Ir‐192	Open field	Eclipse (photons, electrons) Oncentra Masterplan (brachytherapy)	Photons: 1.5cm PMMA above the device, 1cm PMMA plus 3cm of lead under the device Electrons: 0.5cm gel pad over the device, as for photons under the device Brachytherapy: 1cm PMMA topped by another layer of 0.5 cm. The needle to lead the source was positioned at the bottom of the second PMMA layer.	In‐field	Photons, electrons: 2 Gy/day until 60 Gy cumulative dose was reached. Afterwards, 3 fractions of 10 Gy, and 3 fractions of 20 Gy (Cumulative dose of 150 Gy) Brachytherapy: 42 Gy in 6 Gy fractions	Inadequate shock (2 Gy, 12 Gy, 32 Gy photons/48 Gy electrons), Treated VT episode (6 Gy brachytherapy), Loss of telemetry (80 Gy, 90 Gy, 150 Gy photons and electrons) Device detected VT episode (44 Gy photons) Treated VT episode (30 Gy electrons, 12 Gy brachytherapy) Change of program (24 Gy photons, 20 Gy electrons) Random noise (20 Gy, 50 Gy photons) Shock delivery (24 brachytherapy)
56	Gauter‐Fleckenstein et al./ 2022	62 R / ICD	Medtronic; St Jude Medical	Photons/6 MV and 6 MV FFF	Open field	Monaco v 5.11	SSD = 98cm; field size 5 × 5cm^2^; devices placed at distances of 1.0 cm, 2.5 cm, and 7.0 cm from the nominal field edge; 100 Gy at isocenter	Out‐of‐field	3,9 Gy (6MV) at 1cm; 1,5 Gy (6MV) at 2,5 cm; 3,4 Gy (6MV FFF) at 1cm; 1,1Gy (6MV FFF) at 2,5cm; 0,4Gy (6MV FFF) at 7cm	Inappropriate sensing of ventricular tachycardia leading to shock therapy (6MV, 1cm and 6MV FFF at 2,5cm) Inappropriate sensing of atrial or ventricular signals, leading to inhibition of stimulation (6MV FFF at 1cm)
57	Nakamura et al./ 2020	2 PM; 2 CRT (R)	Medtronic, St. Jude Medical	photons/6 MV FFF, 10 MV FFF	Open field	NA	SSD = 100 cm; field 10cm × 10cm; device on top of the phantom	In‐field	4, 6, 8, 10, 12, and 14 Gy/min for the 6 MV FFF beam; 4, 8, 12, 16, 20, and 24 Gy/min for the 10 MV FFF beam	Asynchronous Pacing: 4–14 Gy/min (6 MV FFF), 4–24 Gy/min (10 MV FFF)
59	Falco et al./2021	140 R / 100 PM, 40 ICD	Boston Scientific, Biotronik, Medico, Medtronic, Sorin Biomedica, Sorin/Ela, St Jude Medical	Photons/6MV	Open field	Eclipse, Oncentra Masterplan, Pinnacle	SSD = 96cm, field = 20 × 20 cm^2^, CIED positioned at the centre of a 20 × 23 cm^2^ basin filled with 2 L of physiologic solution (depths 3 cm) placed on a 30 × 30 × 5 cm^3^ RW3 solid phantom	In field	2 Gy, 5 Gy, 10 Gy	Minor V noise (10 Gy) Minor A/V noise (2 Gy, 5 Gy, 10 Gy) Unexpected switch to E.R.I.(2 Gy, 5 Gy) Reset to backup mode VVI/65 (2 Gy) No telemetry (2 Gy) A/V oversensing (2 Gy, 5 Gy, 10 Gy) Inhibition (2 Gy, 5 Gy, 10 Gy) VT/VF detection (2 Gy, 5 Gy, 10 Gy) VT/VF stored (2 Gy, 5 Gy, 10 Gy)
60	Matsubara et al./ 2021	8 PM; 10 CRt (R)	St. Jude Medical, Medtronic	Photons/6 MV	Open field (TBI simulation conditions)	NA	SSD = 100 cm; field 10 × 10cm^2^; device on top of the phantom	In‐field	Accumulated photon dose up to 20 Gy	No malfunction observed up to 20 Gy (100 and 200 cGy/min): shock therapy not activated Inappropriate shock therapies at 10, 20, 40 cGy/min (up to 27.5 Gy accumulated): shock therapy activated
64	Gauter‐Fleckenstein et al./ 2020	4 PM; 64 ICD (R)	Medtronic, St. Jude Medical, Biotronik, Boston Scientific	Photons/6 MV FFF; 10 MV FFF, 18 MV	Open field, VMAT	Monaco v 5.11	3D‐CRT: 40 × 40cm^2^ and device placed at 0–59cm to the beam edge. Dose prescription: 50 Gy for CIED in‐field; 150 Gy if CIED outside the beam. Photo neutron dose measurements at CIED locations with WENDI‐2 FHT 762 with FH 40 G survey meter (Thermo Fischer Scientific, Erlangen, Germany), delivering 10 Gy at the isocenter	In‐field and out‐of‐field	In‐field: Cumulative 50 Gy (TPS evaluation) Out‐of‐field: measured scattered radiation 12–390 cGy Measured photo neutron dose: 67–1310 mSv (10 Gy at isocenter)	5cm, 18MV 3D‐CRT: Reset, Pacing Inhibition, Data Loss ‐ Erroneous Sensing (Vsens), Immediate Shock In field 18MV 3D‐CRT: Shock, Depleted Battery (Delayed) 35 cm, 10 MV FFF VMAT: Erroneous Sensing (Vsens), Pacing Inhibition, Data Loss, Reset (Delayed) 2.5 cm, 10 MV FFF, VMAT Shock, Data Loss

(C) In vivo studies								
Ref	Author/'Year	Study Design	#CIEDs Real (R) / Virtual (V) type	Radiation Type/ Energy	Technique	Irradiation details	CIED Position in‐field/Out‐of‐field	In vivo dosimetry/TPS/Simulation Outcome/Findings
19	Grant et al. /2015	Retrospective study	255 R /123PM, 92 ICD	Photon: 6 MV, 15 MV, 18 MV Electron: 6–16 MeV	3DCRT, IMRT, Gamma knife, Electron therapy.	Abdomen, Brain, Chest, Head/Neck, Pelvis, Total Body. Prescription Dose: 50 Gy, 60 Gy, 70 Gy Fractions: median 15 (range 1–44).	Out‐of‐field	Dosimetry: Direct measurements with TLD‐100 or OneDose MOSFET; TPS estimates used when unavailable, and reference data applied when neither was possible. Corrections accounted for neutron and low‐energy photon overresponse and distance from the field. Outcome: Incident CIED doses reached up to 5.4 Gy without correlation to malfunction. TPS underestimated out‐of‐field doses
58	Aslian et al. /2020	Retrospective study and in vitro	58 R/ 23 PM, 4 ICD, 1 CRT‐D (in vivo) 22 ICD, 8 CRT‐D (in vitro)	Photons: 6 MV FFF (1400 MU/min), 10 MV FFF (2400 MU/min); 6 MV (600 MU/min))	SRS, SBRT, VMAT, 3DCRT	In vivo: Lung and chest, Brain, Liver, Kidney, Spine, Pelvis. Median prescribed dose per fraction: 29 Gy (range 28–54 Gy). Median number of fractions: 3 (range 1–5). In vitro: solid slab phantom with CIED inserted at 1cm depth.	Retrospective: Out‐of‐field In vitro: Inside the RT field at Dmax for rep‐rate tests.	TPS estimation: Mean dose 0.2 Gy (range 0–1.86 Gy). Device response: At 1200–1600 MU/min, transient sensing issues (over‐, under‐, or loss of sensing) occurred without permanent damage or battery change. Beam‐related effects: With 10 MV FFF beams, sensing and parameter changes (mode, threshold, rate, pulse amplitude) arose when CIEDs were partly in the PTV or received <2 Gy outside the field. Shock therapy was deactivated. Monitoring: Continuous supervision with programmers, oscilloscopes, and in‐room cameras; CIEDs positioned with circuitry facing the PTV.
61	Chahid et al. /2024	Case Report	1 R/ PM	Photon: 6 MV FFF	SBRT, VMAT	Lung: Prescription Dose: 48 Gy (12Gy/fr)	Out‐of‐field but close to the field edge.	TPS estimation: CIED dose exceeded 10 Gy. Management: A multidisciplinary approach included precise CIED contouring on planning CT, pretreatment rhythmology assessment, and continuous cardiac monitoring during SBRT. ECG checks were done before and after each session, with device interrogations at 1, 3, and 6 months post‐RT. Outcome: No malfunction occurred.
62	Larsen et al. /2024	Retrospective study	109 R / 81 PM, 28 ICD	Photons: 100 kV, 6 MV, 10 MV 18 MV; Electrons: 6 MeV, 9 MeV, 12 MeV	NA	Head, Head & Neck, Thorax, Thorax & Abdomen, Abdomen, Pelvis, Upper Extremities, Head & Pelvis. Dose: Median 20 Gy (range 0–80 Gy).	Out‐of‐field	TPS estimation: When CT data were available (45.1% of courses), the median CIED dose was 0.23 Gy (95% CI: 0.06–0.94 Gy). Dose distribution: 39.3% of courses received 0–2 Gy, 1.7% 2.1–5.0 Gy, 3.3% 5.1–10.0 Gy, and 0.8% >10 Gy. Management: Two CIEDs were relocated contralaterally; one was reprogrammed. No shielding was applied
63	Hristova/2017	Case report	1 R/ ICD	Photons (6MV)	3DCRT, VMAT, IMRT, TBI	Cranial RT: 24 Gy/12fr; TBI: 8 Gy (2Gy/fr) ‐ Pericardium/Mediastinum: 14 Gy (2 Gy/fr); Mediastinum/Diaphragm): 36 Gy (2 Gy/fr); boost: 10 Gy (2 Gy/fr); bulky recurrence: 46 Gy (2Gy/fr); 2nd Recurrence Mediastinum: 30 Gy (2 Gy/fr)	Out‐of‐field	TPS dose estimation: All plans calculated using CCC and pencil beam algorithms, which have limitations in high‐HU regions (e.g., iron in the device). Maximum cumulative doses: ICD device: 10.07 Gy; electrodes: 68.1 Gy In vivo dosimetry: ICD Dmax: Cranial RT 0.15 Gy; TBI 8 Gy; Mediastinum 0.31 Gy; 1st Recurrence 0.71 Gy; 2nd Recurrence 0.9 Gy Electrodes Dmax: Cranial RT 0 Gy; TBI 8 Gy; Mediastinum 14.8 Gy; 1st Recurrence 20.4 Gy; 2nd Recurrence 24.9 Gy Patient management: High‐risk case. ATAT deactivated during TBI and pericardial RT. Continuous cardiac monitoring with emergency and resuscitation team on standby during treatment.
64	Gauter‐Fleckenstein et al. /2020	Retrospective study and clinical study	200 R/ 108 PM, 92 ICD	Photons: 6 MV, 10 MV, 18 MV, 23 MV; Electrons: 6 MeV Dose rates up to 25 Gy/min for FFF‐IMRT fields	3DCRT, IMRT, IGRT, SBRT	Head, Neck/Thorax Abdomen/Pelvis, Extremities. Dose to CIED: Median 1.02 Gy (range 0–5.37 Gy). Dose to Leads: Median 25.5 Gy (range 1.02–72.7 Gy).	Out‐of‐field. One case of "device in beam" due to patient movement.	Dosimetry: In four AAPM‐group patients, radiation dose at the CIED site was measured with semiflex ion chambers; no direct irradiation occurred. Management: ATAT deactivated; high‐risk pacemaker patients monitored via ECG, and ICD patients via telemetry. Planning: CIED and lead doses calculated in TPS using a Monte Carlo algorithm based on planning CT scans.
65	Luraschi et al/2018	Clinical study	27 V	Electron/6 MeV, 8 MeV, 10 MeV, 12 MeV	IORT with ELIOT	Partial breast irradiation: 21 Gy/1fr at 90% isodose. As boost: 12 Gy	Out‐of‐field TLD distance from the applicator edge: 0.5–7 cm	In vivo dosimetry: TLD‐100 microrods placed in two sterilized catheters in the infraclavicular region. Dose decreased rapidly with distance: < 2 Gy at 2 cm, < 0.5 Gy beyond 2.5 cm. External catheter doses were lower than internal. Radiocromic films confirmed measurements. Recommendation: Maintain at least 2.5 cm distance from the RT field for ELIOT single‐dose treatments.
69	Decker /2024	Clinical study	2 R/ CIED	Photons: 6MV; kV beams for imaging for Cherenkov setup/system.	3DCRT	Breast:40 Gy (2.67Gy/fr); Lymphoma: 20 Gy (4Gy/fr).	Phantom Study: in‐field (30mm), or Out‐of‐field (5mm, 30mm). Patient: Out‐of‐field (about 5 cm distance from field edge.	Dosimetry and monitoring: A Cherenkov iCMOS camera provided real‐time surface monitoring with TPS dose maps for daily setup verification. Custom plastic scintillator discs (1 mm × 15 mm) on the skin over the CIED enabled instant readings; adjacent OSLDs served as references. In vivo dosimetry: Scintillator, OSLD, and TPS doses agreed within 10% in‐field and 5% out‐of‐field. Patient 1: OSLD 0.039 ± 0.007 Gy; Scintillator 0.046 ± 0.002 Gy; TPS 0.042 Gy Outcome: TPS estimates were reliable within 3 cm of the field edge; direct in vivo measurements are advised for 3–10 cm distances
70	Bhimani et al. /2022	Clinical study	2 R/PM	Photons/ 50 kV	IORT	Left breast: 20 Gy/1fr.	Out‐of‐field	Dosimetry: OSLD measurements on the skin near the CIED. Continuous CIED monitoring during surgery and IORT; device located ∼10 cm from the field. Low‐energy beam (50 kVp) used to limit scatter and exposure. Measured doses: Patient 1: 0.23 Gy; Patient 2: 0.185 Gy
81	Hamza /2021	Retrospective study	193 R	Photons (6 MV FF, 10 MV), electrons	3DCRT, IMRT, VMAT, SBRT, Brachytherapy, Electrons, IMPT, GRID	Median RT dose of 50 Gy (range, 7–80 Gy). Treatment sites: Brain, Head and neck, Thorax, Abdomen, Pelvis, Extremity.	Out‐of‐field (14% were <3 cm from the RT field, 34% were 3–10 cm, and 52% were >10 cm.)	Dosimetry: OSLD over the CIED (under 1 cm bolus) during the first fraction; TPS used to estimate maximum dose. Findings: Strong TPS–in vivo correlation for distances >3 cm, weak for <3 cm, with OSLD showing lower doses near the field. Combined TPS and OSLD dosimetry recommended for CIEDs 3–15 cm from the field. Recommendation: Maintain dose rate <0.2 Gy/min; use kV/kV image guidance, apply CBCT cautiously, and minimize jaw settings for MV portal imaging.

(D) Hadron therapy										
Ref	Author/Year	Study Design (in vitro/in vivo/simulation)	# CIEDs Real (R)/Type – Virtual (V)^*^	Device Manufacturer	Radiation Type/Energy	TPS/ Algorithm / Simulation	Irradiation details (set up “in vitro”/target “in vivo studies”/dose prescription)/Simulation details	CIED Position in‐field/Out‐of‐field	Dose to CIED (TPS/in vivo dosimetry)/Secondary neutron Dose	Malfunctions Type
71	Bjerre et al. /2021	In vitro/ simulation	62 R/29 PMs, 33 ICDs	Medtronic, Boston Scientific, St. Jude Medical, Biotronik	PBT (Active Scanning) Range: 161.4 ‐214.7 MeV	Eclipse ver. 13.7 (proton dose); MC simulations with TOPAS ver. 3.2 for neutron dose estimation	A 45 × 30 × 30 cm^3^ PMMA phantom with a cavity on the top for CIED was irradiated at 180°, with 10 × 10 × 10 cm^3^ SOBP and different lateral distances from SOBP edge. Simulated clinical protocol: 72 GyE in 36 fr; CIED simulated with cylinders of 1 cm‐height and 5‐cm diameter at approx device position	Out‐of‐field: cranio‐caudal distance from SOBP edge: 3 cm; lateral distance from SOBP edge: 0.5, 5.0, or 10.0 cm	Secondary neutron dose (H*(10)): 1.91–6.94 mSv	Reset (one permanent lock in safety mode, which required replacement), disabled or loss of RF telemetry, battery depletion
72	Kawakami et al. /2024	In vitro/ simulation	1 V	FPGA: Cmod‐s7 Digilent	CIT (Pencil Beam Scanning) 290 MeV/u	MC simulations with PHITS for neutron flux distributions	Phantom: A 20 × 20 × 50 cm^3^ PMMA with a cavity for FPGA in the middle of the transverse section, at 10 cm from the cranial edge. Irradiation: 1 × 10^9^ particles/s (Gaussian distribution of σ = 11 mm) for 10 min. Secondary particle quality was varied using different shielding materials (PE with ^10^B, Fe, Ni, Pb, graphite plates) between the irradiation position and the FPGA or opposite site of the irradiation position.	Out‐of‐field: distances: 5–20 cm	NA	Memory inversions
73	Okano et al. /2022	In vivo/simulation	20 R/19 PMs, 1 ICD	Medtronic, Boston, St. Jude Medical	CIT (Passive Scattering) Range 220–400 MeV/u	XiO‐N (carbon ion dose); MC simulations with PHITS for neutron distributions and energy spectra	H&N, thorax, liver, prostate, bone and soft tissue 51.6–67.2 GyE in 4–16 fr Simulations: the head was a 10‐cm radius sphere, the body a 10 × 21 × 120cm^3^ cuboid with two 10.5cm semicircular columns filled with water; irradiation with a 10 × 10 cm^2^ field and 6 cm SOBP at 220–290 MeV/u, 1 × 10^8^ particles/s	Out‐of‐field: distances from 95% isodose line to CIEDs: 0 ‐ >30 cm	NA	Premature battery loss, battery power decrease from pre‐treatment baseline
74	Sakai et al. /2024	In vitro	6 to 8 V for each irradiation	FPGA: Cmod‐S7 Digilent	CIT (Passive Scattering) 290, 380 and 400 MeV/u X‐rays 6, 10 and 15 MV	NA	A 30 × 30 × 20 cm^3^ acrylic phantom was irradiated with 5 × 5 cm^2^ and 10 × 10 cm^2^ fields for 10 min. CIT: 4‐cm range shifter; SOBP width set to 6 cm. wobbling method with radius of 8.6 cm to form the fields; intensity of 1 × 10 particles/s X‐Rays: dose rate of 5Gy/min at 10 cm depth	Out‐of‐field: distances from the centre of the phantom: 7.5, 10, 12.5 cm	CIT: 6.2*10^−3^ – 1.6*10^−1^ Gy X‐rays: 0.5 – 4.4 Gy	Memory inversions
75	Seidensaal et al. / 2019	In vivo	31 R/3 ICDs, 28 PMs	Medtronic, St. Jude Medical, Biotronik, Boston Scientific, ELA Medical	PBT ‐ CIT (Active Scanning)	NA	Head, Skull base, H&N, abdomen‐pelvis, thorax / 10–66 GyE, median dose: 51 GyE	Out‐of‐field: distances from PTV: 4.1–17.9 cm (median: 13.4 cm) CIED in beam for 2 patients	Maximum doses restricted to 2 GyE (constraint, neither measured nor calculated)	Enhanced impedance of lead wires, fluctuating during treatment
76	Hashimoto et al/2022	In vivo	69 R/ 64 PM, 4 ICD, 1 CRT‐D	Medtronic, St. Jude Medical, Biotronik, Guidant, Vitatron, Boston Scientific, Sorin Group, NA	PBT ‐ CIT (Passive Scattering) PBT: 115–235 MeV; CIT: 140–400 MeV/n	NA	Liver, lung, prostate, bone and soft tissue, H&N, pancreas PBT: 36.3 to 88.0 GyE with 2.0–6.6 GyE per fr CIT: 28.0–77.0 GyE with /2.2–22.5 GyE per fr	Out‐of‐field: distances (lung patients): 0–25 cm	NA	Transient reset Over‐sensing
77	Ueyama et al. /2016	In vivo, in vitro, simulation	7 R/ 7 PM	Medtronic, Boston Scientific, St. Jude Medical	PBT (Passive Scattering) 150 e 210 MeV	NA	Before treatment, a phantom study using the same PM model as that implanted in patients, to measure the secondary neutron dose and compare it to the simulations Clinical protocols: patient 1: lung/66 GyE in 10 fr patient 2: pancreas/50 GyE/ in 5 fr other patients: prostate, larynx, lung	Out‐of‐field: distances from field to generator: 13‐>50 cm; one patient with partial lead irradiation	Secondary neutron dose: measures were within the estimated values at simulation Measured: 96.4–154.6 mSv; calculated: 103.2–158.4 mSv	Reset to VVI mode (safety back‐up program)
78	Yoshida et al. /2023	In vivo	4 R/ PM	Medtronic, Biotronik, St. Jude Medical	PBT (Passive Scattering) 220 MeV	NA	Prostate/74 GyE in 37 fr	Out‐of‐field: distances from the treatment isocenter: 54–56 cm	NA	Mode change from DDD (60 bpm) to DDI (70 bpm) ("back‐up mode"); prolongation of RR intervals

(E) TPS accuracy									
Ref	Author/Year	Study Design (in vitro/in vivo/simulation)	# CIEDs Real (R)/Type ‐ Virtual (V)*	Radiation Type/Energy	Technique	TPS/algorithm	In vivo dosimetry/Irradiation/ Treatment/Simulation details	CIED Position in‐field/out‐of‐field	TPS Accuracy/Other outcomes
42	Sung et al. /2019	In vivo	3 R/NA	Photons /NA	VMAT, SBRT	Eclipse system (ver. 13.7) /Progressive Resolution Photon Optimizer (PO) and Acuros XB (AXB)	In vivo dosimetry using OLSDs detectors placed on the skin near the CIED for three patients Calculated mean doses obtained from the TPS in a contoured region similar to the measured area	In‐field, out‐of‐field	TPS‐calculated out‐of‐field and surface doses were up to five times lower than measured doses, reflecting large TPS uncertainties (e.g., up to 55% underestimated dose at 11.25 cm from the field edge)
45	Aslian et al. /2021	In vitro/ simulation	1 V	Photons/6MV, 15 MV Secondary neutrons	Open Fields	Elekta Monaco V.5.11.01/X‐ray Voxel‐based MC (XVMC) for photon dose MCNPX version 2.7 for photon and neutrons simulations	Photon dose was calculated (TPS), simulated (MCNPX), and measured (microDiamond, PinPoint 3D, Semiflex 3D detectors) at 1 cm, 2 cm (CIED depths), and 10 cm (reference depth) CIED relative neutron damage quantified using the methodology suggested by Ezzati and Studenski (82–83)	Out‐of‐ field	XVMC consistently underestimated out‐of‐field doses, average errors of ‐17% (for <10 cm) and ‐31% (10–16 cm). Using 15 MV photons amplified the underestimation by up to 11%. The relative neutron damage is 36% less than the damage reported in the literature for an 18 MV photon beam.
66	Yan et al. /2018	In vivo/in vitro	In vivo: 3 R/NA In vitro: 1 V	Photons /6MV	Open Fields, 3DCRT	Eclipse/Analytical Anisotropic Algorithm (AAA) v13.6	In vivo. OSLD on the skin, near the CIED, with/without 1–2 cm bolus and with/without 2 mm lead on the bolus In vitro. Use of a parallel plate chamber (Markus) in a solid water phantom to measure doses with/without 1–2 cm bolus covering the chamber	In vivo: <5 cm from the field edge In vitro: out‐of‐field	TPS underestimate the dose to CIED, but it predicts the CIED dose more accurately when bolus is used In vivo dosimetry showed 20%–60% lower dose with bolus Bolus reduced chamber‐measured dose at 0.5–1.5 cm depth by ∼7%–48%
67	Bourgouin et al. /2015	In vitro	1 V	Photons/6 MV, 23 MV	3DCRT, IMRT	Pinnacle3 version 9.6 /NA	PSD (Exradin W1) used to measure the doses in a torso phantom at a CIED ‐equivalent depth (1.5 cm), for 10 clinical RT plans (H&N, Lung, Breast, and Nodes), both with and without a 1.6 mm lead shield PSD measurements to build a simple, empiric model to estimate the dose The TPS was used to evaluate the dose at the location of the CIED and to measure the distance between the 50% isodose line and the CIED	Out‐of‐field	On average, the difference between the TPS prediction and the measurement was 71% and only 14% for the dose prediction model Mean dose reduction with lead shield: 19%±13%; for breast cases: 31%±15%
68	Bourgouin et al. /2019	In vitro	1 V	Photons/6 MV, 23 MV; Electrons/6 MeV,18 MeV	Open fields	Pinnacle3 version 9.6/NA	Measurements in water tank with a PSD (Exradin W1) detector, with shielding on top to evaluate the shielding efficiency of a thermoplastic‐wrapped lead foil PSD measurements were compared with dose calculations from a commercial TPS	In/out‐of‐fields	TPS poorly models out‐of‐field dose at distances greater than 5 cm, leading to an underestimation of the dose magnitude. Lead shielding reduced the CIED dose (at 0.5 cm depth, 3–15 cm out‐of‐field) by around 52% for electron/6 MV photon beams and up to 71% for 23 MV photon beams
79	Peet et al. /2016	In vitro	1 R/ PM	Photons/6 MV	Helical Tomotherapy, open fields, VMAT, 3D‐CRT	Eclipse/AAA; Oncentra/CCC; Tomotherapy/Planning Station	PM installed in an anthropomorphic phantom (Rando). Radiochromic film and OSLDs to measure the dose in the vicinity of the device during the delivery of square fields and clinical treatment plans	In/out‐of‐field	TPS substantially underestimated dose (factors of 3–10 for lung/chest wall plans) Skin dose could be greater or lesser than dose at device depth, depending on treatment site. Square field reference data can be used as an upper estimate for absorbed dose near a CIED in complex, multi‐angle treatments
80	Delana et al. /2020	In vitro	1 R/ PM	Photons/6 MV	Open Fields, 3DCRT, IMRT	Elekta Monaco v.5.11/ XVMC; Elekta Oncentra Masterplan v.4.5.3/CCC; Philips Pinnacle3 v.9.8/CCC; Philips Pinnacle3 v.9.10/CCC	In vivo dosimetry using TLD‐100 and Mosfet (TN‐RD‐70‐W) detectors above PM PM embedded in Real Water3 phantom. with its proximal edge from 1 cm to 10 cm from the field edge at 2 cm depth Dose calculation by the TPSs to evaluate the dose at the location of the CIED	Out‐of‐field (ranging from 1 cm to 10 cm) and close proximity	TPS‐to‐TPS Comparison: TPS dose differences were higher for IMRT (approx 3.5%) than square beams (2%). Dose calculation depends on the algorithm (XVMC higher than CCC) rather than the Linac commissioned TPS‐to‐Measurement Comparison: Three out of four TPSs agreed well with square beam measurements within 4 cm of the field edge, but only one maintained accuracy with the modulated beam. Over /underestimations for modulated beams up to +20% to ‐40%

(F) Monte Carlo simulations						
Ref	Author / Year	Radiation Type / Energy	Software	Simulation objectives	Irradiation/Simulation/Treatment details	Simulation Outcomes
41	Stick et al. / 2022	PBT Active scanning/Energy range: 139–199 MeV	TOPAS v3.5	Estimate Secondary Neutron Dose to CIEDs for patients with breast and H&N cancer undergoing PBT to calculate the ambient dose equivalent H∗ (10) from secondary neutrons Establish Clinical Decision Algorithms for patient selection based on results for the maximum secondary neutron dose to the CIED, using a threshold of 7 mSv/fr, beam energy, CIED‐CTV distance, the distance between the CIED and the 5% and the 25% isodoses, the size of CTV, dose‐fractionation schedule (only for breast cancer), and CTV risk, intermediate or low (H&N cancer)	Proton therapy plans with fixed Relative Biological Effectiveness (RBE) of 1.1 and a range shifter of 57 mm water equivalent thickness Breast: 50 Gy RBE (2 Gy RBE/fr), 40 Gy RBE (2.67 Gy RBE/fr), and 26 Gy RBE (5.2 Gy RBE/fr). H&N: 66 or 68 Gy RBE to CTV high risk, 60 Gy RBE to CTV intermediate risk, and 50 Gy RBE to CTV low risk in 33 or 34 fr. Adjacent to target (contralateral or ipsilateral for breast, ipsilateral for H&N) After simulation, the doses were imported to the Eclipse TPS ver. 13.7 for evaluation	For patients with breast cancer and with contralateral CIED, the secondary neutron dose to the CIED was below 7 mSv/fr for CTV *<* 1500 cm^3^ in 2 Gy/fr and CTV *<* 1000 cm^3^ in 2.67 Gy/fr. The secondary neutron dose to the CIED was below 7 mSv per fraction for all patients with H&N cancer
82	Ezzati et al. /2017	Photons/18 MV	MCNPX 2.7	Quantify the relative neutron damage induced in CIED	Damage was assessed using silicon damage response functions (specifically the updated Norgett‐Robinson‐Torrens or NRT factors) and ICRP neutron dose conversion factors (ICRP‐74) Damage parameterized as a function of field size, off‐axis distance, and depth by normalizing to central axis damage at phantom surface	Damage probability related to the neutron equivalent dose rather than fluence. Increasing the depth to the CIED or the central axis distance reduces the probability of damage. As field size increases, the region of high damage probability extends to a greater distance beyond the edge of the field than with smaller fields At an off‐axis distance >50 cm or a depth > 10 cm, the probability of damage is < 10% of the central axis damage probability for all field sizes. Using posterior beams or treating sites distant to the CIED will minimize the probability of damage.
83	Ezzati et al. /2019	Photons (>10 MV)	MCNPX 2.7	Design of gantry‐mounted neutron applicators, attaches to wedge/electron applicator mount to reduce the probability of neutron damage Model Used: a previously validated Linac model (Ref [16]) was utilized to design and optimize the neutron applicators	Materials considered: Polyethylene and borated polyethylene (0%, 3.5%, 5%, 20% boron weight composition) Various applicator thicknesses (2, 4, 6, 8, 10 cm) and outer dimensions. Applicator Mass: Fixed at 10 kg. Damage Calculation: The probability of neutron damage in CIEDs was calculated using silicon damage response functions and the method employed by Ezzati et al. (82)	The neutron applicators were found to reduce the probability of damage to the CIED by up to a factor of 3.4, depending on the off‐axis distance The optimal applicator design was the one with a thickness of 4 cm and a boron composition of 3.5% in borated polyethylene. The applicator also reduced the in‐field damage probability up to 170%.
84	Kakino et al/ 2021	Photons /15MV	PHITS (v. 3.14)	Investigate the performance of neutron‐shielding sheets (NSS) in reducing photoneutron‐induced damage to CIEDs Measure the γ‐ray and leaking X‐ray dose from NSS Analysis of neutron damage with and without the NSS by changing NSS length and thickness	NSS performance valuated by comparison between experimentally measurements using a Eu:LiCaAlF6 (LiCAF) scintillator and simulations Prompt γ‐rays and leaking X‐rays measured with EBT3 GafChromic films Neutron Relative Damage evaluated using Ezzati et al. model (82)	Comparison between MC simulation and experiment agreed within a 30% with some exceptions Doses below 5 cGy/1500 MU for Prompt γ‐rays and leaking X‐rays Damage rate reduced by 43% when a 2‐cm‐thick bolus and a rubber silicone sheet with 80% B4C were applied
85	Mele et al/ 2023	Photons/ 15 MV, 18 MV	MCNP code (v. 6.2)	Develop and test detailed MC geometries of the LINACs used to create reliable computational tools for further investigation into neutron contamination. Evaluate the thermal neutron fluence in CIED region from conventional radiotherapy treatments (multicentric study) and compare it to the critical value of about 10^9^ cm known to cause CIED damage Evaluate the fast neutron fluence	Neutron fluence was simulated (MCNP) and measured (CR‐39 with B4C converter, TLD pairs (7LiF:Mg,Ti and 6LiF:Mg,Ti), bubble detectors for fast component) Measurements: detectors inside a BOMAB‐like phantom mimicking the patient's trunk and positioned in the cardiac region simulating typical CIEDs position. Doses ranging from 20 to 60 Gy. MU delivered at isocenter for measurements	The MC simulations of thermal neutron fluence are consistent with the passive detector measurements at all centres, validating the accuracy of the complex accelerator and treatment vault models. Thermal neutron fluence comparable to the critical value (e.g., 1.75 × 10^52^MU for 18MV, 20MU). Fast neutron fluence of 10^4^ cmMU (vertebra) and 10^23^ cmMU(iliac wing).

(G) Imaging							
Ref	Author/ Year	Topic	Study design (In vivo / in vitro)	# CIEDs real (R)/Type‐ virtual (V)^*^	Imaging system considered	Output Indicator	Key outcome
43	Georgiu et al /2020	CIED movement during RT	In vivo	12 R/6 PM, 6 ICD	Varian's On‐Board Imaging (OBI) system	Horizontal, vertical and rotational movement of CIED position in 206 daily image to planning image	The mean change in position of a CIED during a patient's treatment course is <1mm for almost all patients, the day to day variations were up to 5mm.
86	Aslian et al / 2018	Imaging dose from two imaging system	In vitro	NA	Elekta XVI and Varian OBI kV‐CBCT systems	Dose to CIED from different CBCT imaging systems and protocols	If the CIED is positioned inside of the FOV the dose contribution from the daily positioning CBCT might not be negligible and should be taken into account for CIED risk stratification.
87	Odlozilikova et al. / 2018	Impact of metal artifact deletion algorithm (MDT) on dose calculation near CIED	In vitro	9 R/5 PM, 4 ICD		Impact of MDT on plan parameters and CIED dose	With MDT it was easier to contour CIED, calculated dose differences with and without the algorithm were up to 3% of the prescription dose (in breast plans).
88	Ese et al. /2018	Impact of different Hounsfield Unit range and metal artifacts reduction (MAR) algorithm in simulation CT	In vitro	1 R ICD, 1 titanium disc, 1 copper disc, 1 open cut empty titanium IPG case	Siemens Somatom Definition Flash Dual Source CT Scanner. Helical mode acquisition.	HU values of metallic objects	The extended HU range better represents the electron densities of metal objects. The MAR algorithm underestimates such densities.

(H) MRI‐related considerations and MR‐guided Radiotherapy							
Ref	Author/ Year	Topic	Study design (In vivo / in vitro)	# CIEDs real (R)/Type‐virtual (V)^*^	MRI related considerations (simulation)	MR guided RT	Key outcome
89	Yang et al/2022	MR‐guided Radiotherapy and 1.5T MR‐linac	In vivo	3 R/ 3 PM	CIED safety by exposing patients to multiple MRI scans in a short period. Treatment simulations were performed on a CT‐simulator and with a 1.5 T MR‐simulator.	3 rectum and prostate SBRT treatments. In vivo measurement with EBT film for devices placed 3–10 cm from the field edge. MRI scans were performed using the standardized T2W sequence.	Local safety protocol on CIED management in MRgRT was developed, rigorously executed, and demonstrated on four patients On the MR‐Linac: Dose estimation based on Out‐of‐field dose data.
90	Keesman et al./2024	MRgRT on a 1.5 T MR‐linac,	In vivo	11 R / 9 PM, 2 ICD	Evaluated MRI and RT workflows for MR‐conditional CIEDs. Focused on the impact of device‐induced artifacts on image quality and automated gating performance.	Adaptability of standard MRgRT workflows to CIED patients was assessed. Investigated geometric accuracy of tumor tracking and the feasibility of continuous cardiac monitoring during treatment.	Demonstrated that 1.5 T MR‐linac treatments are feasible and safe for CIED patients. Real‐time monitoring and adapted workflows ensured treatment accuracy without compromising device integrity.
91	Gach et al./2019	SBRT using a 0.35T MRI Linac in patient with ICD	In vivo	1 R / ICD	Real‐time tumor tracking and beam gating were performed using 2D sagittal cine TrueFISP imaging with a 3 mm static gating boundary. The "null band artifacts" caused by the ferrous components of the ICD influenced the selection of the tracking target	Although the ICD manufacturer classified 0.35 T MRI as "off‐label" the medical physics team cleared the device for MR‐IGRT based on dose estimates and low‐field risk assessments.	The procedure was completed successfully without safety incidents, demonstrating the feasibility of low‐field (0.35 T) MRI‐guided radiation therapy
92	Mayinger et al./2020	MR‐guided cardiac radioablation (STAR) on a 0.35T	In vivo, in vitro	1 R / ICD	ICD leads caused local signal voids and image distortions on 1.5 T MRI. Safety was ensured through multidisciplinary protocols, including device reprogramming to asynchronous mode and continuous monitoring.	Single‐fraction SBRT (25 Gy) delivered on a 0.35 T MR‐Linac. Automated gating was used based on a 2D cine‐MRI (sagittal view), though significant artifacts required careful selection of the tracking window.	Successful feasibility study of MR‐guided cardiac radioablation. No ICD malfunctions or interference with the therapy were observed. The low‐field MR‐Linac allowed for precise targeting despite the presence of the electronic device.

#### 3.2.1 LVAD devices

Studies on LVADs in RT are scarce, mostly case reports about a limited number of devices. They covered the effects of direct/indirect irradiation, dose calculation accuracy, and imaging artifacts. LVADs are typically kept outside the radiation field,^48^, ^49^, ^50^, ^51^, ^52^, ^53^ following maintenance guidelines, even for nearby irradiation sites.^52^ However, both in vitro^54^ and in vivo^51^ direct photon irradiation studies have shown that LVAD pumps can withstand exposure (e.g., 10 MV at 6.8 Gy;^54^ 16 MV at 1.2 Gy during TBI with pump shielding^51^) without any malfunction.

While direct photon irradiation of the LVAD controller (6MV at 50 Gy in single fraction) caused no malfunction in vitro,^48^ direct proton irradiation poses risks. An in vitro study^44^ found that LVAD pump and driveline handled fractionated doses (70–75 Gray‐Equivalent (GyE)), but a 30 GyE single fraction caused functional issues (low‐flow alarm, communication loss), likely due to secondary neutrons. Even if there is no general agreement about the shielding of implanted electronic devices in the literature, in case of high dose proton treatments, the authors of this study suggest this possibility as a preventive measure. It could be inferred from theoretical CIED malfunction occurrence in scattered neutron fields. Device intensive monitoring in high‐dose proton therapy is also suggested. Clinical cases that avoided direct LVAD irradiation^49^, ^50^, ^53^ also reported no malfunction. Authors generally highlight careful patient selection^49^ and management,^51^ individual safety checks,^48^ and multidisciplinary treatment planning.^49^, ^50^, ^51^

Conventional opposed field TBI with high energy photons^51^ is a particularly challenging treatment for LVAD patients, but specific precautions can reduce the dose to the device components.

LVADs introduce major planning complexities, including planning Computer Tomography (CT) imaging artifacts^52^, ^54^ that hinder accurate delineation and influence Hounsfield Unit (HU)‐electron density and dose calculation, often requiring multimodality imaging, and careful beam angle selection to avoid the device.^54^

#### 3.2.2 PM and ICD devices

##### Photon and electron RT

*In vitro studies*. Recent studies have emphasized the influence of DR and beam energy on CIED behavior, particularly with FFF beams. Gauter‐Fleckenstein et al.^55^ reported immediate damage only during direct irradiation or with 10 MV WFF beams, while a subsequent study^56^ found that ICDs positioned within 7 cm from the beam edge exhibited transient (local dose rate (LDR) > 6.6 cGy/min) or persistent errors (LDR > 46.8 cGy/min) during 6 MV FFF‐Volumetric Modulated Arc Therapy (VMAT). They suggested that the LDR may be the primary cause of ICD malfunction rather than the cumulative dose, and hypothesized a safety threshold of 6 cGy/min (or a distance of 7 cm from the field edge). Nakamura et al.^14^, ^57^ observed immediate, but reversible pacing inhibition during direct irradiation at DR > 800 MU/min (in 75% of 6 MV FFF and 100% of 10 MV FFF devices), with 10 MV FFF causing cumulative damage, including battery depletion at 10 Gy and telemetry failure by 134 Gy. Similarly, Aslian et al.^46^ reported 50% inappropriate sensing and anomalies with 10 MV FFF at 1200 MU/min and only 25% with 6 MV FFF beams, suggesting caution when using 10 MV FFF in pacing‐dependent patients near the Planning Target Volume (PTV).

Beam energy further influences direct irradiation outcomes. Zecchin et al.^15^ and Mollerus et al.^16^ observed no ICD malfunctions with 6 MV photons up to 130 Gy, indicating notable CMOS resistance, while Zaremba et al.^17^ demonstrated that 18 MV beams (30 Gy) caused 14 malfunctions in five devices compared to only one malfunction at 150 Gy with 6 MV. Falco et al.^40^, ^58^ found 99.3% of CIEDs free of post‐irradiation errors with 6 MV up to 10 Gy, with permanent damage limited to non–MRI‐compatible PMs at ≥2 Gy. Matsubara et al.^59^ confirmed the safety of 6 MV beams in TBI with shock therapies disabled, and Baehr et al.^47^ extended this analysis across modalities (6 MV photons, 6 MeV electrons, and ^1^ ^9^ ^2^Ir brachytherapy), identifying modality‐specific risks: photons and brachytherapy causing inadequate shock, and electrons inducing random noise.

Regarding photoneutron effects, which are strongly energy‐dependent, Zecchin et al.^18^ showed 15 MV high‐energy treatments caused neutron‐induced malfunctions in 52% of ICDs and 13% of PMs, even without direct exposure. Gauter‐Fleckenstein et al.^55^, ^56^ found photon beams ≥10 MV caused failures from scattered neutrons in CIEDs at off‐axis positions, while 6 MV FFF‐VMAT remained safe at distances ≥ 7 cm from the field. A significant reduction in neutron damage—36% less for 15 MV photons compared to 18 MV literature data—was effectively demonstrated by Aslian et al.^45^ through the use of MC simulations.

*In vivo studies*. Clinical incidence of CIED malfunctions may differ across cases. Aslian et al.^46^ found no malfunctions outside FFF beams (6 MV and 10 MV) in patients, although their phantom study suggests avoiding 10 MV FFF when possible in pacing‐dependent patients or with devices close to the PTV. Chahid et al.^60^ similarly reported no malfunctions in a PM receiving > 10 Gy from 6 MV FFF SBRT, when careful patient assessment was performed to optimize outcomes; they emphasized the critical role of interdisciplinary collaboration and continuous cardiac monitoring to prevent device malfunction.

Grant et al.^19^ observed a 7% overall CIED MR, with all malfunctions occurring in neutron producing beams (15MV and 18MV photons), and with ICDs more susceptible than PMs. Conversely, Larsen et al.^61^ reported no device failures or lead parameter changes during or after RT in 109 patient records, provided appropriate precautions were taken. Hristova et al.^62^ described a patient undergoing TBI with 8 Gy to the ICD; it functioned normally despite a cumulative dose > 10 Gy (TBI and consolidation RT). This higher tolerance was attributed to the large Source‐to‐Surface Distance (SSD) and extremely low DR in TBI.^62^ The authors note that with very low DR, CIEDs can withstand direct irradiation at doses exceeding guideline limits.

Gauter‐Fleckenstein et al.^63^ reported a 17.9% reduction in CIED malfunctions after adopting the DEGRO/DGK guideline compared with earlier AAPM recommendations, confirming the guideline's effectiveness—especially the use of 6 MV photons and risk‐based management—in preventing radiation‐induced CIED failures.

Accurate CIED dose estimation relies on in vivo dosimetry using various detectors, including Optically Stimulated Luminescent Dosimeter (OSLD),^42^ Thermoluminescent Dosimeter (TLD) and radiochromic films,^64^ ionization chambers,^65^ and Plastic Scintillator Detector (PSD).^66^, ^67^ These measurements also serve to compare experimental data with TPS‐calculated doses, as discussed in Section 3.2.3. Real‐time monitoring with Cherenkov imaging and scintillation dosimetry provides complementary solutions.^68^

For IORT (TARGIT‐IORT, ELIOT) in breast cancer, in vivo dosimetry is crucial. Bhimani et al.^69^ reported very low PM doses (∼0.2 Gy) during TARGIT‐IORT measured with OSLD, considering it safe, but calling for further distance studies. Luraschi et al.^64^ used TLDs in the infraclavicular region, the typical CIED site in breast cancer patients during electronic IORT (ELIOT), and showed that a 2.5 cm safety distance between the radiation applicator and the CIED ensures a dose below 2 Gy at the device location.

##### Hadron therapy

Secondary neutrons from Hadron Beam Therapy (HBT), that is, Proton Beam Therapy (PBT) and Carbon Ion Therapy (CIT) can cause CIED malfunctions, particularly SEUs, thus classifying HBT as high‐risk technique by guidelines (e.g., AAPM, AIFM).^28^, ^29^, ^38^, ^39^

*In vitro studies*. In vitro experiments and MC simulations help define risk thresholds. Bjerre et al.^70^ irradiated 62 CIEDs with pencil beam scanning protons, finding the probability of device resets increased by 55% per mSv of secondary neutron dose, depending on the CIED–Spread Out Bragg Peak distance. Significant battery depletion occurred in five devices, though no sensing errors or inappropriate therapies were observed in 362 fractions. In MC simulations, Stick et al.^41^ reported neutron doses < 7 mSv/fraction, consistent with Bjerre's limits, and proposed eligibility algorithms based on dose, CIED–PTV distance, beam path, and clinical target volume. Kawakami et al.^71^ found neutron fluence as the primary driver of SEUs in CIT, while protons and heavy fragments caused ≤30% of soft errors at 400 MeV/u. All above mentioned articles use active scanning beam techniques. MC simulations showed that passive scattering CIT induces more soft errors in FPGA circuits than < 10 MV X‐rays (see Supplementary Material S2). Error rates increase at higher energy and smaller fields (5 × 5 cm^2^) likely due to increased neutron generation caused by carbon ion interaction with the collimator (5 × 5 cm^2^ vs. 10 × 10 cm^2^). Conversely, rates decrease with distance from the field (7.5–12.5 cm). Results emphasize caution when using 400 MeV/u CIT.^72^, ^73^

*In vivo studies*. Clinical data among the considered studies show variable outcomes. Seidensaal et al.^74^ reported no malfunctions in 31 patients treated with active scanned protons/carbon ions (median dose 51 Gy, mean CIED–PTV distance 13.4 cm) even with devices in the beam path. Conversely, Hashimoto et al.^75^ observed six transient resets among 47 PBT patients, but none in 23 CIT cases, with ICDs more affected than PMs. Similarly, Okano et al.^72^ found no SEUs in 20 CIT patients, though three had early battery depletion (none requiring immediate replacement), possibly due to device‐to‐95% isodose line distance > 30 cm, which reduced neutron exposure. Case reports highlight the unpredictability of high‐dose exposure. Ueyama et al.^76^ reported CIED resets in two PBT patients exposed to high neutron doses (range: 96.4–154.6 mSv), although both were asymptomatic. Yoshida et al.^77^ described two prostate PBT cases using 220 MeV passive protons with PM mode changes (e.g., backup mode activation, oversensing) and attributed these to the higher neutron dose in passive scattering compared with active scanning. All in vivo device malfunctions occurred with passive scattering PBT/CIT.

#### 3.2.3 Modelling radiotherapy effects

##### TPS accuracy

TPS dose prediction for CIEDs varies by algorithm, measurement technique, and device position. Yan et al.^65^ found good agreement between TPS and in vivo/in vitro measurements using OSLD/Markus chamber in Three‐Dimensional Conformal Radiation Therapy setups (> 2 cm depth, 1–2 cm bolus, 2–5 cm from field edge), while other studies identified systematic underestimation of out‐of‐field doses, especially in VMAT and Intensity‐Modulated Radiation Therapy (IMRT), requiring finer tuning for accurate out‐of‐field calculations.^78^, ^79^ Errors can reach 71% (PSD, Exradin W1).^66^, ^67^ To improve out‐of‐field accuracy, these researchers proposed an exponential decay model, reducing discrepancies to 14% (without shielding) and 33% (with shielding). Lead shields lowered dose by 23%, though weight and scatter remain issues. Consistent TPS underestimation (e.g., Monaco X‐ray Voxel‐based Monte Carlo, XVMC^45^) supports the need for in vivo dosimetry, particularly in VMAT.^42^ Combined TPS and in vivo OSLD dosimetry remains comparable up to 15 cm,^80^ a longer range than the 10 cm recommended by the AAPM TG‐203.^29^

##### MC simulation

Neutron‐induced damage to CIEDs is a major concern in high‐energy photon radiotherapy. MC and experimental studies have investigated neutron production and fluence, and mitigation strategies. Key findings show neutron damage correlates with dose (not just fluence),^81^ and while the background neutron exposure is relatively isotropic, large fields increase scatter and expand the damage region.

Mitigation efforts have included borated polyethylene applicators (effective, but heavy) and neutron shielding sheets,^82^ which use a water‐equivalent bolus and a B4C silicone sheet to achieve a 43% reduction in CIED failure.

Mele et al.^84^ confirmed thermal neutrons dominate within the body during 15/18 MV treatments, with high fluence near the heart, and fast neutrons decrease with distance.

For proton therapy, Stick et al.^41^ confirm feasibility by using a 7 mSv secondary neutron dose threshold (estimated as ambient dose equivalent)^70^ to guide patient selection, provided target volumes remain below specific limits (1500 cm^3^ for 2 Gy/fraction or 1000 cm^3^ for 2.67 Gy/fraction).

#### 3.2.4 Imaging in radiotherapy

Georgiou et al.^43^ retrospectively analyzed daily kV radiographs in 12 lung patients, observing mean displacements of 2 mm (max 3.5–5 cm) and rotations of 2° (max 43°) throughout treatment, potentially impacting TPS predictions. Aslian et al.^85^ found CBCT doses up to 1.77 cGy per scan (∼50 cGy over 30 fractions) for CIEDs within the Field Of View (FOV); out‐of‐field devices received negligible doses.

CT metal artifacts can produce dose underestimation (∼3% near CIEDs and pacing leads^86^), causing dose inaccuracies near high‐Z materials.^54^ Extended HU ranges can better represent metallic implants for dose calculations, but Metal Artifact Reduction (MAR) algorithms may underestimate HU values, requiring further investigation.^87^

#### 3.2.5 MRI‐related considerations and MRI‐guided radiotherapy

The integration of MRI into radiation oncology workflows has introduced specific challenges and opportunities for patients with CIEDs.

The implementation of MRI‐guided workflows for radiotherapy treatments necessitates a multi‐institutional framework to maximize the advantages of real‐time guidance while minimizing the risks associated with electromagnetic interference as suggested by Yang et al.^88^ and Keesman et al.^89^ Risk management strategies for MRI‐Linac include adherence to safety thresholds of specific absorption rates below 2 W/kg and gradient slew rates within established values (e.g., <80 mT/m/s) to prevent electrode heating or unintended stimulation.^90^ While the vendor's MRI specifications are limited to clinical 1.5 T and 3 T systems, the use of CIEDs at 0.35 T has proven successful despite its off‐label status. Mayinger et al.^91^ and Gach et al.^90^ reported no device damage or safety incidents with low‐field MRI‐Linac; in this context, it is fundamental that each procedure was preceded by a comprehensive risk assessment and clearance from medical physicists.

Clinical integration further requires standardized, cross‐disciplinary protocols involving radiation oncologists, medical physicists, and cardiologists to optimize cardiac device programming—such as utilizing asynchronous modes for pacing‐dependent patients—both preceding and following each treatment fraction.^88^ Another topic to consider in the presence of CIED is the monitoring of the geometric accuracy; the systematic use of B_0_‐mapping is indispensable for quantifying spatial distortions, in order to ensure the geometric accuracy required for accurate dose calculation and target localization.^90^ In addition, cardiac monitoring and synchronization challenges must be addressed. Given that conventional electrocardiogram (ECG) signals are inherently compromised by interference within the MRI‐Linac environment, current evidence supports the transition to wireless peripheral pulse oximetry as a more robust solution for real‐time cardiac monitoring and synchronization during advanced procedures, including STereotactic Ablative Radiotherapy (STAR).^91^

#### 3.2.6 Evidence‐based workflow for medical physicists

Based on the reviewed data, an evidence‐based flowchart (Figure 4A–E) was developed to guide medical physicists in treatment planning and risk assessment for patients with CIEDs. The tool outlines key physical parameters and safer management strategies (green boxes), from CT imaging and contouring (Figure 4A) to beam selection and simulation, with device‐specific workflows for LVAD and PM/ICD (Figure 4B–D) and MRI‐Linac specific workflow (Figure 4E), informed by malfunction statistics across hadrons, photons, and electrons.

FIGURE 4Flowchart of management, risks and warnings to be considered during radiotherapy treatment of patients with CIEDs. (a) Pre‐treatment management section; (b) LVAD specific consideration and possible risks; (c, d) PM / ICD specific considerations, possible risks and malfunction rates. (e) MRI‐Linac specific considerations. MR = Malfunction Rate. The green rectangles in the flowchart refer to safer treatment conditions. *For accurate in vivo dosimetry measurements optically stimulated luminescent dosimeters (OSLD^42^), thermo luminescent dosimeters (TLD), radiochromic films,^64^ Plastic Scintillator Detectors (PSD),^66^, ^67^ and ionization chambers^65^ are suggested by the selected literature.”

**Figure:**
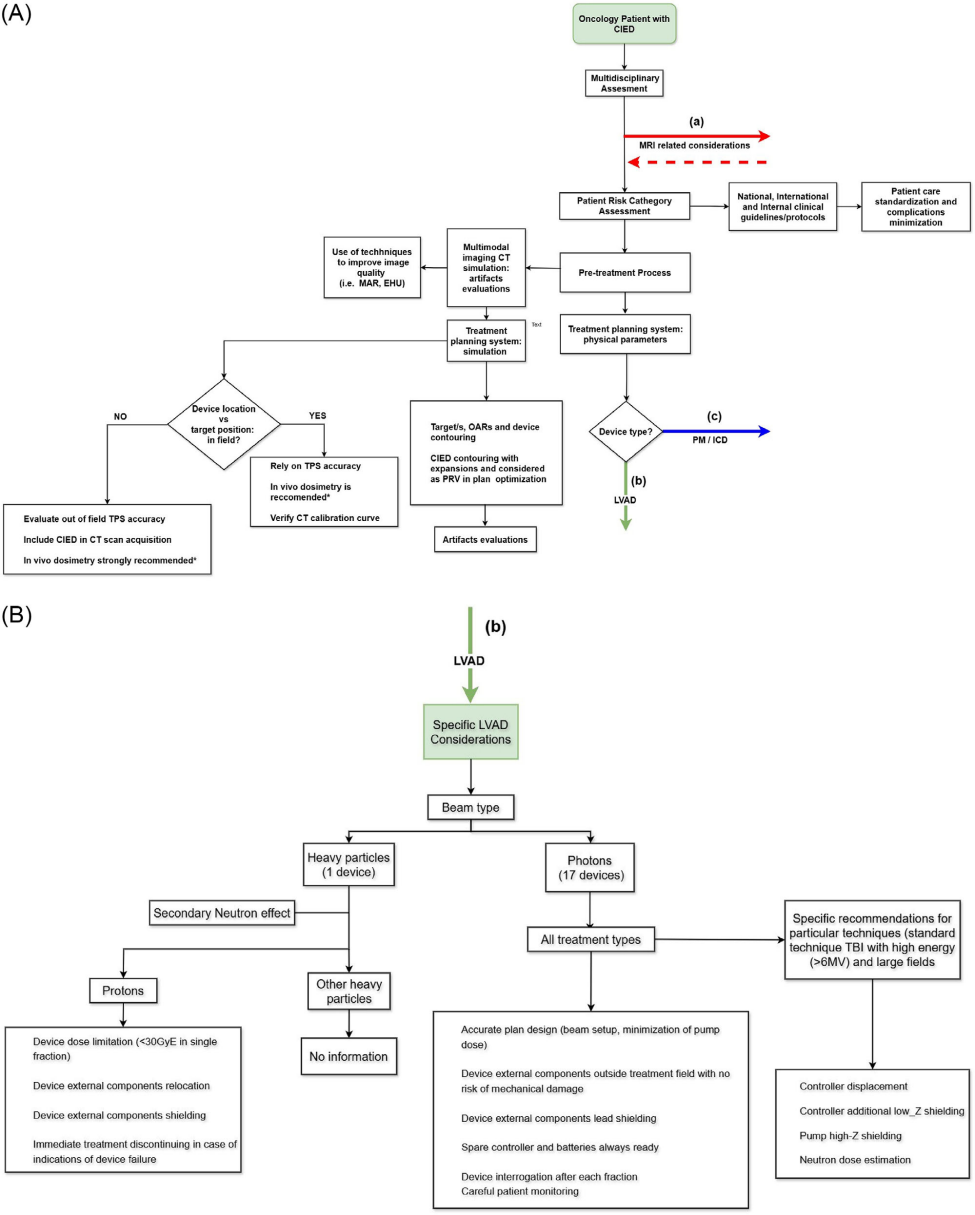

**Figure:**
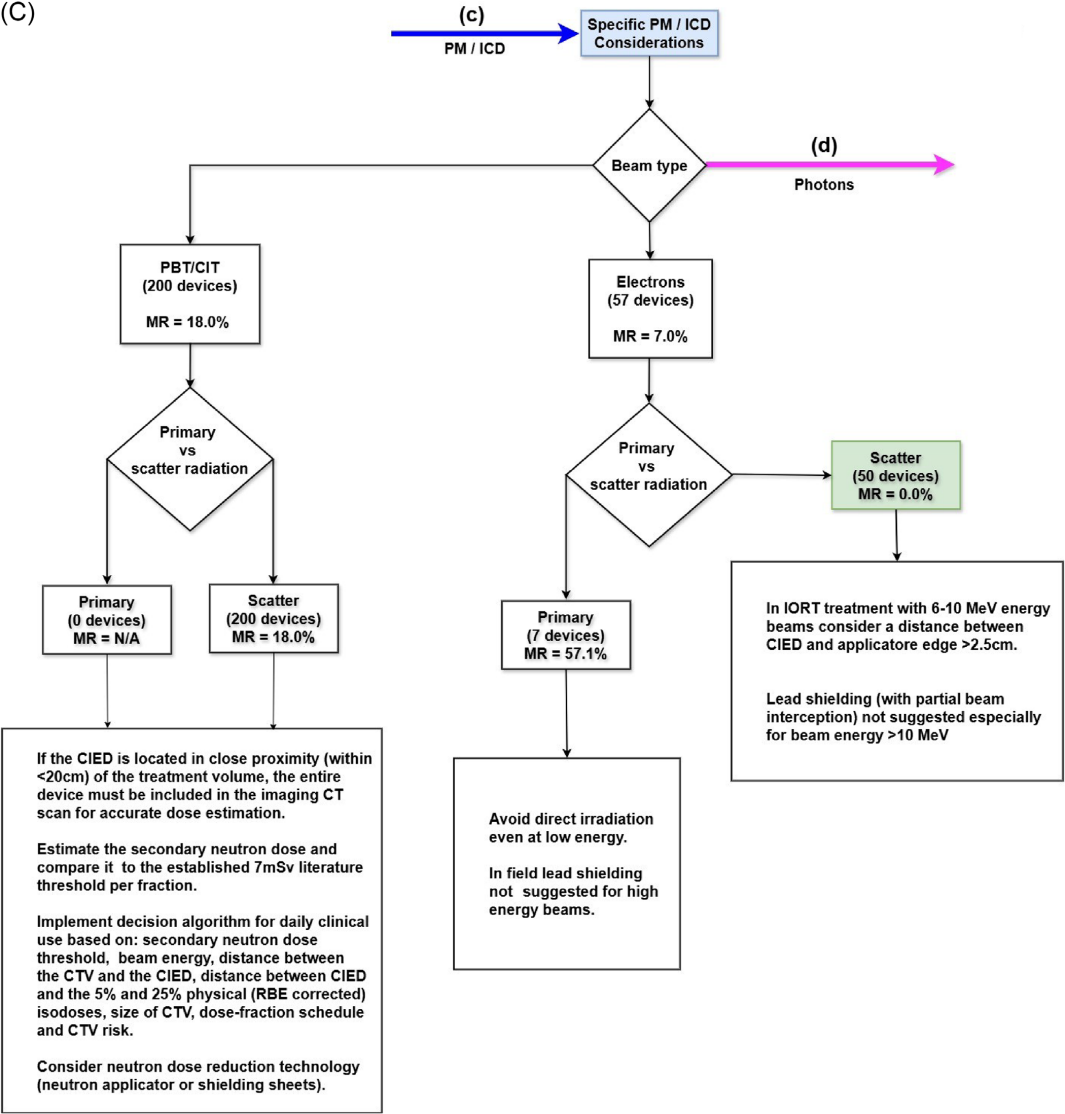

**Figure:**
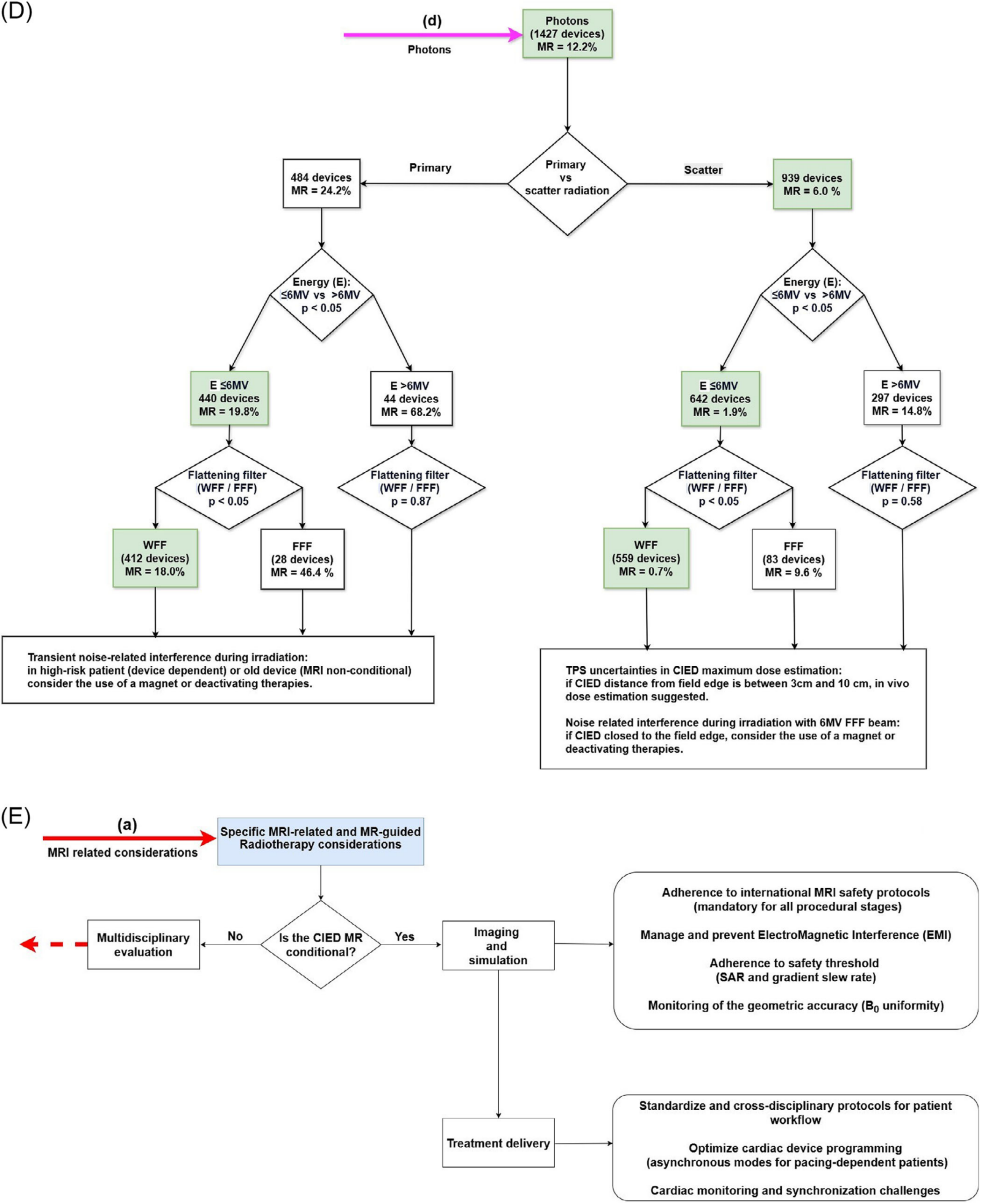

## 4 DISCUSSION

Radiotherapy risk for CIEDs is determined by a complex interaction between radiation physics and semiconductor architecture. Modern CIEDs utilize nanometric CMOS nodes (65 nm–45 nm), which present a physical paradox: thinner oxide layers provide greater resistance to TID, yet increase susceptibility to SEU. TID induces permanent hardware degradation by generating electron‐hole pairs within CMOS insulation, resulting in increased leakage currents and threshold voltage shifts. Modern nanometric nodes frequently exhibit resilience up to 10 Gy, as their thinner oxide layers facilitate charge tunneling and reduce charge trapping. Despite this inherent resilience, these smaller components are increasingly sensitive to SEUs, making historical 2 Gy limits less reliable than a risk‐based monitoring approach. Moreover, high‐voltage ICD components are more sensitive than those in PMs, rendering them more vulnerable to environmental stress and high‐dose‐rate beams (such as FFF beams), which can induce transient photocurrents. These currents may mimic cardiac signals, leading to dangerous oversensing or pacing inhibition. Therefore, a management strategy that prioritizes real‐time device behavior over simple cumulative dose tracking is required.^13^, ^14^, ^15^, ^16^, ^17^, ^18^, ^19^, ^20^, ^21^, ^22^, ^23^, ^24^, ^25^, ^26^, ^29^, ^36^ Further details are provided in Supplementary Material S2.

To bridge the gap between these physical principles and clinical outcomes, this review analyzes 1769 real and several virtual devices—over half of which were irradiated in vivo—to clarify failure mechanisms and patient risks. The findings confirm that while software malfunctions are the most frequent complication (12.6%), their underlying causes vary significantly by beam type and energy. Specifically, malfunction rates are higher for neutron‐producing beams (i.e., high energy photons or hadron beams) compared to 6MV photon beams. Based on statistical analysis and literature, the CIEDs WG team developed flowcharts (see Figure 4A–E) summarizing key main findings and providing basic guidance for medical 6 physicists.

A crucial preventive measure is a multidisciplinary pre‐treatment meeting involving all specialists to classify the patient according to a risk protocol consistent with national and international guidelines. Proper classification ensures safe management throughout the radiotherapy process. Many studies confirm that, despite inherent risks, rigorous management of CIEDs, continuous monitoring, and adherence to updated guidelines—such as the DEGRO/DGK 2015 recommendations,^30^ which helped eliminate an 18% complication rate—significantly reduce failures.^60^, ^61^, ^62^, ^63^, ^80^, ^92^

Following Figure 4A, during pre‐treatment CT simulation, attention should be given to artefact reduction to improve image quality and dose accuracy through advanced correction or imaging methods.^54^, ^86^, ^87^, ^93^ Device motion may cause uncertainty in the dose received due to the dynamic nature of CIED positioning.^43^ Such displacement can affect TPS‐calculated maximum dose, potentially misclassifying patient risk. Applying a geometric margin to the CIED during contouring (i.e., defining a CIED Planning Organ at Risk Volume (PRV) as per ICRU Report 83) is advisable to account for uncertainty during treatment optimization.

TPS accuracy is essential for managing CIED patients and reducing risk. Device position and distance from the beam critically affect dose estimation. Out‐of‐field dose calculations vary among TPSs,^42^, ^45^, ^65^, ^78^, ^79^ showing both over‐ and underestimation, especially with modulated beams.^79^ A simplified prediction model^66^ and external validation^42^, ^45^, ^65^, ^78^, ^79^ improve accuracy of the absorbed dose received by CIEDs for IMRT and VMAT beams. In vivo dosimetry using OSLDs,^42^ TLDs,^64^ ionization chambers,^65^ or PSDs^66^, ^67^ is strongly recommended,^42^, ^43^, ^80^ particularly during IORT, to minimize risk while ensuring safe field‐edge distances.^64^, ^69^ A lead‐thermoplastic shield can reduce cumulative dose, particularly for out‐of‐field or high‐energy photons but caution is required with high energy electron beams, as an interception of the shield might increase the dose to the CIED that can be underestimated by the TPS.^67^ Real‐time monitoring systems^68^ can further improve safety. ICDs mainly exhibited transient detection issues, while older PM models were more vulnerable to permanent damage.^40^, ^58^, ^75^

### 4.1 LVAD

In LVADs (Figure 4B), the external controller and power supply are especially sensitive to radiation;^94^ hence they should be positioned outside the field and, if necessary, shielded.^94^, ^95^ Moreover, spare controllers, batteries, and an expert team should be available for emergencies.^94^, ^95^ Modern LVADs include sensors and memory storage in the pump, requiring extra precautions.^94^ Treatment planning should minimize pump dose and correct for metal artifacts.^54^, ^94^ Patient monitoring and device interrogation after each fraction aid early fault detection. Additional care is needed with high‐energy photons and large fields.^51^ In this specific case, controller displacement and low‐Z shielding may assist with exposure reduction, while avoiding neutron generation; if the pump is within the field, high‐Z shielding may be applied. Treatment simulation measurements, in vivo dosimetry and neutron dose evaluation with proper equipment are advised.^51^

Data on hadron‐induced LVAD effects remain limited, and only concerning protons.^44^, ^95^ Dose limitation is strongly advised, especially for direct single‐fraction exposure. Since protons generate more secondary neutrons than photons,^44^, ^95^ relocation or shielding of external components may be prudent.^44^, ^50^ Treatment should be immediately suspended if malfunction occurs.^44^, ^50^

### 4.2 Hadron therapy

Patients receiving PBT or CIT are considered high‐risk, mainly due to secondary neutron production. The primary risk here is the SEU, a stochastic mechanism where a single heavy particle or neutron interaction with the semiconductor lattice causes a state change in the memory^15^, ^18^, ^19^, ^25^ (more details are reported in Supplementary Material S2).

Primary particles can also directly cause CIED failures.^29^, ^38^, ^39^ These beams can induce malfunctions even when CIEDs lie outside the treatment field.^81^ AAPM TG‐203 notes that cumulative dose alone does not damage hardware if outside the beam; the primary risk is software malfunction and neutron‐induced SEUs. These stochastic “bit flips” occur independently of total absorbed dose. When detected, CIEDs perform safety resets, reverting to backup mode‐a protective, but clinically concerning event. The risk correlates with neutron dose and differs by manufacturer.^41^, ^70^, ^81^ All in vivo malfunctions occurred with passive scattering beams that have a higher neutron production than active scattering techniques.^72^, ^75^, ^76^, ^77^

Across studies, a MR value of 18% was found among 200 analyzed devices under scattered radiation. CR malfunctions, observed in both in vitro and in vivo settings, were mostly permanent (95.2%) rather than transient (4.8%) considering both PBT and CIT. Malfunctions with active scanning techniques were reported only in one in vitro study, where devices were positioned at 0.5 cm to the beam edge;^70^ otherwise malfunctions were observed only in passive scattering as opposed to active scanning due to greater neutron yield.^29^, ^70^, ^75^, ^76^, ^77^ Proton‐induced malfunctions were primarily software‐related (e.g., data/memory resets).

For CIT, only one study reported hardware‐related malfunctions (i.e., permanent component damage);^72^ others observed no malfunctions,^41^, ^44^, ^73^, ^74^ attributed to strict dose control and safety clinical protocols. These include maintaining a safety distance,^72^, ^73^ estimating neutron dose below the 7 mSv threshold,^41^, ^70^, ^84^ and using neutron‐reduction technologies like specialized applicators or shielding sheets.^82^, ^83^ Recommendations appear in Figure 4C.

### 4.3 Electron therapy

Few studies^19^, ^46^, ^61^, ^63^, ^64^, ^67^ addressed electron treatments, especially above 10 MeV, limiting conclusions. A significant difference in MR between primary and scattered conditions was found (Figure 4C).

Only three studies provided suggestions:^47^, ^64^, ^67^ direct irradiation should be avoided, even at low energies, to minimize dose and damage. A distance of at least 2.5 cm from the applicator edge in IORT (6–10 MeV) ensures dose < 2 Gy. Lead shielding may be used with low‐energy beams but avoided at higher energies to prevent Bremsstrahlung. Partial or lateral shielding must be carefully evaluated to avoid excess scatter. Clinical use of >10 MeV beams are rare due to alternative techniques; significant neutron production should not be a concern with electron beams due to the lower neutron cross‐sections for (e^−^, n) reaction for electrons compared with photons. Consequently, little information exists on high‐energy electron use in CIED patients.

### 4.4 Photon therapy

Photon‐related risks are multifactorial, influenced by energy and DR in both direct and scattered exposure.

As shown in Figure 3, software errors (60%) predominate (the 86% of which being transient), while the main hardware fault is battery depletion. Transient interference during Linac operation may be clinically relevant depending on pacing dependence.

The CIED–target distance is the primary factor affecting function: MR differed significantly between in‐field and scattered exposure (*p* < 0.05) (Figure 4D), supporting guideline recommendations to avoid direct irradiation.^29^, ^30^, ^31^ While risk classification is dose‐based, analysis revealed MR–dose correlation only when all devices were pooled; separately, no clear dependence emerged, suggesting direct exposure is more harmful than cumulative dose.

Evidence supports current guidelines recommendations to use non‐neutron‐producing energies (≤6 MV), which are markedly safer than higher energies in‐field and scattered conditions. When higher energies are necessary, strict adherence to protocols and intensive monitoring is essential both during and after radiotherapy as recommended by international guidelines.^29^, ^30^

The impact of the DR is particularly significant; a high nominal DR (> 8 Gy/min) in the treatment field, such as that found in FFF beams, can induce transient photocurrents within the device circuitry.^14^, ^57^ These radiation‐induced currents can mimic cardiac signals (oversensing), leading to temporary pacing inhibition during irradiation.^14^, ^57^ While these effects typically resolve after beam cessation, they represent a critical physical mechanism that necessitates real‐time monitoring for pacing‐dependent patients.

In complex 6 MV FFF plans, transient or persistent errors may occur inside or near the field,^14^, ^55^, ^56^, ^57^ though MRI‐conditional CIEDs show greater resistance even at high doses.^17^, ^40^, ^60^, ^62^

For 10 MV FFF where secondary neutrons predominate as the main cause of malfunctions, statistical analysis revealed no significant differences in MR between primary and scattered radiation. Transient errors occurred in FFF plans inside or close to the field (< 5 cm)^46^, ^57^ and therefore particular care should be paid for high‐risk device dependent patients, for example considering the use of a magnet or deactivating therapies before irradiation. The only observation of such errors far from the field occurred at isocenter doses > 100 Gy.^55^ Key recommendations are summarized in Figure 4C.

### 4.5 Specific MRI‐related and MRI‐guided radiotherapy considerations

The integration of MRI‐guided workflows (MRI‐Linac) represents a paradigm shift for CIED patients, moving from absolute contraindication to proactive risk management. Adherence to international MRI safety protocols remains mandatory throughout all procedural stages.^96^ While CIEDs were once precluded MRI access, the current challenge for medical physicists lies in characterizing ‘gray zones’, such as mixed‐brand systems lacking combined safety testing despite having MRI‐conditional components.^89^, ^97^, ^98^

In this context, the physicist's role expands to include geometric accuracy. Metallic leads and generators induce B_0_ inhomogeneities and susceptibility artifacts that can compromise dose calculation and target localization, factors marginal in CT‐guided workflows.^90^ Consequently, B_0_‐mapping and robust sequences are essential for high‐precision treatments such as STAR.^91^

Additionally, the MRI‐Linac environment necessitates a re‐evaluation of patient monitoring.^90^, ^91^ The failure of conventional ECG due to electromagnetic interference requires physics‐led protocols and alternative tools, such as wireless peripheral pulse oximetry, for both safety and cardiac gating.^88^ Ultimately, safe MRI‐Linac treatment of CIED patients demands intensified synergy between physicists, cardiologists, and radiation oncologists to balance online guidance benefits with MRI‐related complexities.^88^, ^98^ Key recommendations are summarized in Figure 4E.

## 5 CONCLUSIONS

In conclusion, managing cancer patients with CIEDs during radiation therapy is a complex undertaking that requires strong physics expertise and a collaborative multidisciplinary approach. Several factors must be considered: the use of high‐energy or neutron‐producing beams, DR, the proximity of the device to the treatment field, artifacts managements, TPS accuracy, MRI related challenges and the specific device type and generation (ICDs are more sensitive than PMs, and older models are more vulnerable).

However, serious malfunctions are rare when rigorous management protocols, based on up‐to‐date guidelines and comprehensive monitoring (including careful patient selection, meticulous planning, and continuous device monitoring), are followed.

From this perspective, the developed flowcharts are intended to serve as practical decision‐support tools for the community of medical physicists working in radiotherapy, who are involved in managing, within their scope of competence, the safety of patients with CIEDs.

## AUTHOR CONTRIBUTIONS

**Maria Daniela Falco**: Conceptualization, Supervision, Visualization, Writing—original draft, Writing—review & editing **Tiziana Malatesta**: Conceptualization, Data curation, Supervision, Visualization, Writing—original draft, Writing—review & editing **Agnese Barbareschi**: Conceptualization, Data curation, Formal analysis, Supervision, Visualization, Writing—original draft, Writing—review & editing **Emilio Mezzenga**: Conceptualization, Data curation, Supervision, Visualization, Writing—original draft, Writing—review & editing **Rita Alaimo**: Conceptualization, Data curation, Formal analysis, Supervision, Visualization, Writing—original draft, Writing—review & editing **Anna Delana**: Formal analysis, Visualization, Writing—original draft, Writing—review & editing **Mara Severgnini**: Visualization, Writing—original draft **Maria Grazia Brambilla**: Writing—review & editing **Stefano Andreoli**: Visualization, Writing—original draft **Stefano Ren Kaiser**: Visualization, Writing—original draft **Marco Mapelli**: Visualization, Writing—original draft **Eugenia Moretti**: Visualization, Writing—original draft **Rita Consorti**: Visualization, Writing—original draft **Maria Mormile**: Visualization, Writing—original draft **Giulia Rambaldi Guidasci**: Visualization, Writing—original draft **Lidia Strigari**: Visualization, Writing—original draft

## FUNDING INFORMATION

The authors declare that no funding was received for this work.

## CONFLICT OF INTEREST STATEMENT

The authors declare that they have no known competing financial interests or personal relationships that could have appeared to influence the work reported in this paper.

## Supporting information




Supporting information

Supporting information

## Data Availability

All data are reported in the manuscript and in supplementary materials.
