# Supplementary material for: Systematic review of physical and dosimetric criteria for managing CIEDs in radiotherapy: A medical physicist's perspective
Source: J Appl Clin Med Phys. 2026 Aug 16;27(8):e70740. doi: 10.1002/acm2.70740 (PMC13478559; doi:10.1002/acm2.70740)
Supplement: Supplementary file 1 [file ACM2-27-e70740-s002.docx]

Participant:
Affiliation:

**Article**	
Article ID	*[#]*
Reference	*[Author, Title, Journal]*
Year	*[Year]*
Professionals Involved (Authors)	*[Physicist / Radiotherapist / Cardiologist /Electrophysiologist / other]*
In Vivo Study	*[Yes/No]*
In Vitro Study	*[Yes/No]*
Simulation (e.g., MC)	*[Yes/No]*
Meta-Analysis	*[Yes/No]*
Other Study Type	*[Specify]*
**CIED (Cardiac Implantable Electronic Device) Details**	
N. CIEDs Considered	*[#]*
Pacemaker (PM)	*[#]*
Cardiac Resynchronization Therapy (CRT)	*[#]*
Implantable Cardioverter Defibrillator (ICD)	*[#]*
Left Ventricular Assist Device (LVAD)	*[#]*
CIED Explanted	*[Yes/No/Not Applicable (NA)]*
Electrodes Attached	*[Yes/No/NA]*
Manufacturer & Model	*[Manufacturer/Model / NA]*
Shock Therapy Deactivated	*[Yes/No/NA]*
CIED Settings	*[Pacing Mode | Pacing Rate (bpm) | Sensing Threshold (mV) | Pacing/Sensing Polarity | Other Settings / NA]*
Notes on CIED	
**Radiotherapy System and Technique**	
RT System/Device	*[Linac/Tomotherapy/Cyberknife/Passive Scattering (Protons/Ions)/Pencil Beam Scanning (Protons/Ions)/NA]*
Irradiation Beam	*[Photons/Electrons/Protons/Other Charged Particles (Specify)/NA]*
RT Technique	*[VMAT / IMRT / 3D / NA]*
Particle Technique	*[SFO* / MFO* / Other / NA]*
Setup Imaging Type	*[MV / kV CBCT / EPID / NA]*
Setup Imaging Dose Evaluation	*[Yes/No/NA]*
Notes on RT System	
**Planning and Dose Parameters**	
Treatment Planning System (TPS)	*[Software name and model]*
Calculation Algorithm	*[PBC / AAA / AXB / CCC / XVMC **/ Other*
Prescribed Dose	*[Total Dose / # fractions / NA]*
Cumulative Dose	*[cGy / NA]*
Cumulative Dose Evaluated On	*[CIED Case, Electrodes (Leads), Surface (with/without Bolus), Other (Specify), NA]*
Photon Energy With Flattening Filter (WFF)	*[MV/NA]*
Flattening Filter Free Beams Used (FFF)	*[Yes/No/NA]*
Energy photons FFF	*[MV/NA]*
Energy (Non-Photon Beams)	*[MV/NA]*
Charged Particles Type/Delivery	*[Passive Scattering / Active Scanning / NA]*
Neutron Production	*[Yes/No/NA]*
Dose Rate (MU/min)	*[Min, Max / NA]*
Notes on Planning	

**Irradiation Strategy and Monitoring**	
CIED Irradiated	*[Primary/Scattered/NA]*
Out-of-Field Irradiation	*[Yes/No]*
CIED Monitoring (Oscilloscope/Load/Other)	*[Yes/No/NA]*
CIED Simulator Used	*[Yes/No/NA]*
Magnet Use During Irradiation	*[Yes/No/NA]*
Device Reprogramming	*[Yes/No/NA]*
Device Monitoring During Exposure	*[Yes/No/NA]*
In Vivo Dosimetry	*[Yes/No/Instrument used]*
Notes on Strategy	
**Irradiation Setup and Phantom**	
Source-Surface Distance (SSD)	*[cm / NA]*
Field Size	*[cm^2 /NA]*
CIED Distance from Field Edge/Target	*[cm / NA]*
CIED Depth	*[cm / NA]*
Notes on Setup	
Phantom Used	*[Yes/No/NA]*
Water Equivalent Phantom	*[Yes/No/NA]*
Water Phantom	*[Yes/No/NA]*
Anthropomorphic Phantom	*[Yes/No/NA]*
Experimental/In-House Phantom	*[Yes/No/NA]*
Notes/Phantom Description	
**Target Site**	
Chest or thorax	*[#]*
Breast	*[#]*
Head and Neck	*[#]*
Other Target Site	
**Device Malfunctions & Outcome**	
**Malfunctions investigatet?**	*[Yes/No]*
**If yes:**	
Online Malfunctions found ***	*[#/NA]*
Malfunctions post irradiation found	*[#/NA]*
Clinically relevant malfunctions	*[#]*
**Malfunctions duration:**	
Permanent	*[#]*
Transient	*[#]*
Software errors	*[#]*
Hardware errors	*[#]*
Mixed (hardware/software) errors	*[#]*
Notes on Malfunctions	
**Study Highlights/Recommendations on:**	
Treatment Planning Strategies (Algorithm, CT-ED Curve, Dose, etc.)	*[Yes/No/NA]*
Irradiation Parameters (Photons/Particles, Energy, Dose Rate, etc.)	*[Yes/No/NA]*
Imaging (Setup or Simulation)	*[Yes/No/NA]*
Use of Magnet	*[Yes/No/NA]*
Device Shielding	*[Yes/No/NA]*
Device Monitoring	*[Yes/No/NA]*
CIED Settings	*[Yes/No/NA]*
Other Recommendations	*[Specify]*
Additional Information	

*SFO = Single Field optimization, MFO = Multi Filed Optimization
** PBC = pencil beam convolution, AAA = anisotropic analytical algorithm, AXB = Acuros XB, CCC = Collapsed cone convolution, XVMC = Monte Carlo
*** Online Malfunctions: Refers to functional anomalies (e.g., noise, inhibition, telemetry loss) occurring simultaneously with beam-on. Detection of such transient effects is highly dependent on whether active device monitoring was performed during the irradiation procedure.
