# Supplementary material for: Systematic review of physical and dosimetric criteria for managing CIEDs in radiotherapy: A medical physicist's perspective
Source: J Appl Clin Med Phys. 2026 Aug 16;27(8):e70740. doi: 10.1002/acm2.70740 (PMC13478559; doi:10.1002/acm2.70740)
Supplement: Supplementary file 1 [file ACM2-27-e70740-s001.docx]

The mechanisms of failure in Cardiac Implantable Electronic Devices (CIEDs) during radiotherapy (RT) are primarily driven by the interaction of ionizing radiation with the device's electronic components, particularly modern Complementary Metal-Oxide-Semiconductor (CMOS) technology. Modern CIEDs use smaller semiconductor nodes (e.g., 65nm or 45nm), which are physically more sensitive to Single Event Upset (SEU) but more resistant to Total Ionizing Dose (TID). This has improved battery life but increased vulnerability to specific physical failure mechanisms [13-26,29,36]. Failures are categorized into three primary physical processes based on how radiation interacts with the device's hardware and software:

#### 1. Charge Trapping in Dielectrics (Total Ionizing Dose, TID)

This mechanism results in permanent hardware damage caused by the total amount of radiation absorbed by the device over the entire treatment course [13-14, 16-17, 20-21, 23-24,29].

- **The Mechanism:** Ionizing radiation creates electron-hole pairs within the silicon dioxide (SiO_2_) insulation layers of the CMOS circuits. While electrons are mobile and exit the oxide, positive charges (holes) become trapped in the oxide interface.
- **The Physics:** This leads to increased leakage currents and shifts in the threshold voltage of the transistors.
- **Variation Factor:** Malfunction rates vary based on the dose rate and the specific manufacturing node (the size) of the chip, as thinner oxides in modern devices react differently to charge trapping.
- **Clinical Outcomes:** These changes can lead to premature battery depletion, "runaway" pacing, or a complete loss of pacing and sensing capabilities.
- **Risk Thresholds:** While historical guidelines suggested a strict 2 Gy limit, modern data indicates that permanent failures are unpredictable; some devices show degradation at low doses, while others tolerate significantly more. Although the AAPM TG-203 [29] describes that the risk of component drift and excessive battery drain increases with increasing cumulative dose, modern in vitro studies and meta-analyses have observed a lack of correlation between device malfunction and incident dose up to 10 Gy [19,40,59].
- **Lead Vulnerability:** While the generator is the primary target of radiation, the cables represent the system's 'Achilles heel.' Although leads are generally resistant to ionizing radiation, high-voltage components and insulation can be stressed by the chemical and mechanical environment. Furthermore, any pre-existing lead fragility may exacerbate the clinical impact if radiation alters the generator's sensing thresholds.

#### 2. Transient Photocurrents (Dose Rate Effects)

High-energy pulses from a Linear Accelerator (LINAC) can generate instantaneous ionization. Unlike cumulative damage, these effects are temporary and occur only while the radiation beam is actively being delivered. They are dependent on the instantaneous intensity of the radiation (Gy/min) [20-21, 23-24, 26].

- **The Mechanism:** A high-flux pulse of radiation (especially from Flattening Filter Free beams) creates a massive, transient concentration of charge carriers in the semiconductor’s depletion regions.
- **The Physics:** This induce photocurrents within the device's sensing circuitry that can mimic cardiac signals or cause "latch-up"—a state where the device draws excessive current, potentially leading to a permanent short circuit..
- **Clinical Outcomes:**
  - **Oversensing:** The CIED misinterprets radiation-induced noise as natural cardiac electrical activity. This can lead to pacing inhibition (critical for pacemaker-dependent patients) or the delivery of inappropriate shocks by ICDs. Systematic meta-analysis indicates that ICDs are significantly more vulnerable to these types of malfunctions than pacemakers.
  - **Telemetry Failure:** Temporary loss of communication between the device and the programmer during irradiation.
  - **Lead Noise:** If a lead already has sub-clinical insulation defects [21], it may be more susceptible to environmental EMI or radiation-induced noise, leading to diagnostic errors.
- **High-Risk Factors:** Techniques using FFF beams deliver dose at much higher rates (up to 24 Gy/min), significantly increasing the probability of these transient sensing errors [13-14,29].
- **Meta-Analysis Data:** Studies show that ICDs are significantly more likely to be affected by these malfunctions than pacemakers (OR 2.75)[23].

#### 3. Single Event Effects (Neutron-Induced Resets)

These are stochastic (random) events caused by secondary neutrons. They are considered the most dangerous because they can occur even if the CIED is located far outside the primary radiation field [15,18-20,23,25,29].

- **The Mechanism:** When using photon energies > 10 MV or proton therapy, neutrons are generated through nuclear interactions. These neutrons cause displacement damage, physically knocking silicon atoms out of their lattice positions. Furthermore, thermal neutrons can be captured by Boron-10 (often used in chip passivation), triggering an alpha-particle emission.
- **The Physics:** **Single Event Upsets (SEUs)**. A single particle can flip a bit in the device’s memory (RAM or registers), i.e. an accidental change of a binary 0 to a 1
- **Variation Factor:** This explains why malfunctions (like resets or parameter changes) occur more frequently at higher beam energies (> 10 MV), where neutron production is physically possible.
- **Clinical Outcomes:**
  - **Soft Errors:** Minor software glitches or loss of diagnostic data.
  - **Power-On Reset (Safety Mode):** The device undergoes a full software reset and reverts to factory "Safety Settings" (e.g., VVI pacing at 65 bpm). This disables all personalized programming and life-saving ICD therapies until the device is manually reprogrammed [19,23]
- **Predictors:** Beam energy is the most significant predictor. Beam energy >10 MV significantly increasing the risk of reset [36] found a Relative Risk of 9.98 for malfunctions when using high-energy beams, and Karimzad et al. [22] confirm that software resets remain the most frequent clinical complication in modern practice.

All these considerations are integrated in the following Table.

### Summary of CIED System Vulnerabilities

| **Component** | **Mechanism** | **Nature** | **Primary Risk** | **Key Reference** |
| --- | --- | --- | --- | --- |
| **Generator (CMOS)** | Cumulative Dose | **Permanent** | Battery drain and depletion, hardware failure | 13-14, 16-17, 20-21, 23-24,29 |
| **Circuitry** | Dose Rate / Electromagnetic Interference (EMI) | **Transient** | False sensing, inhibited pacing, oversensing | 20-21, 23-24, 26 |
| **Memory (RAM)** | Neutrons (SEU) | **Software** | Reset to Safety Mode/Power on reset | 15,18-20,23,25,29 |
| **Leads** | Mechanical/Env. Stress | **Mechanical** | Insulation failure, proarrhythmia | 20-21 |
